# Pre-agricultural intensification of plant use in Pleistocene Sri Lankan rainforests

**DOI:** 10.1038/s41559-026-03082-6

**Published:** 2026-05-20

**Authors:** Nicolas Bourgon, Marcus Oelze, Noel Amano, Oshan Wedage, Nimal Perera, Patrick Roberts

**Affiliations:** 1 Department of Coevolution of Land Use and Urbanisation, Max Planck Institute of Geoanthropology, Jena, Germany; 2 Bundesanstalt für Materialforschung und-prüfung, Berlin, Germany; 3 Max Planck Institute of Geoanthropology, Jena, Germany; 4 Department of History and Archaeology, University of Sri Jayewardenepura, Nugegoda, Sri Lanka; 5 Excavation Branch, Department of Archaeology of the Government of Sri Lanka, Colombo, Sri Lanka

**Keywords:** Ecology, Archaeology

## Abstract

Tropical rainforests have often been considered marginal environments for Pleistocene hunter-gatherers, yet archaeological research in Sri Lanka demonstrates long-term occupation of these habitats from ~48,000 years ago (ka). Material evidence indicates specialized hunting of arboreal mammals, as well as the use of plant resources, but plant consumption is often difficult to detect because organic remains preserve poorly in rainforest settings. Here we present zinc isotope data (*δ*^66^Zn) from Late Pleistocene to Late Holocene human (*n* = 24) and faunal tooth (*n* = 57) enamel, spanning ~20–3 ka of rainforest occupation in Sri Lanka. Our results show that humans consistently occupied an intermediate trophic position, indicating mixed diets of animal and plant foods. Over time, human *δ*^66^Zn values shift towards those typical of herbivores, suggesting an increasing reliance on plant resources. This pattern predates the regional introduction of crop agriculture and indicates that rainforest foragers were intensifying plant use long before farming emerged.

## Abstract (web summary)

Isotopic data from Late Pleistocene to Late Holocene human and faunal tooth enamel in a Sri Lankan rainforest reveal mixed human diets throughout the sequence, with a trend towards intensifying plant consumption, even before the regional introduction of crop agriculture.

## Main

**Fig. 1:**
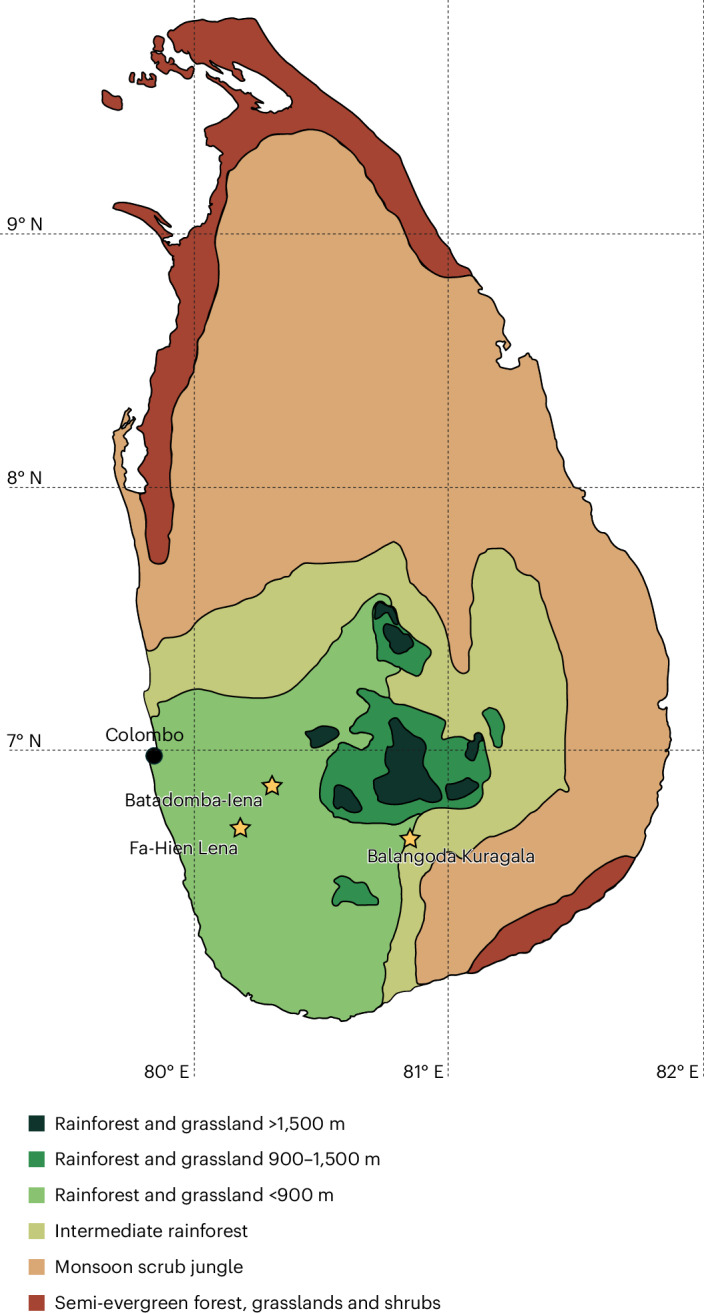
Vegetation zones and location of studied archaeological sites in Sri Lanka (Balangoda Kuragala, Batadomba-lena and Fa-Hien Lena). The Wet Zone (rainforest and grasslands) lacks a significant dry period and supports elevation-structured tropical rainforests; the Intermediate Zone (intermediate rainforest) has a short, muted dry season and transitional forest structure; and the Dry Zone (monsoon scrub jungle and semi-evergreen forest, grasslands and shrubs) has a distinct dry season dominated by monsoon forest and scrub jungle. The island’s vegetation zones were redrawn on the basis of published data from Gunatilleke et al. (2005)^85^ and Erdelen (1988)^86^.

In recent years, the notion that tropical rainforests were inhospitable^1,2^ and ecological barriers to Pleistocene human dispersals^3–5^ has been challenged by the increasing evidence of occupation and adaptation to these environments by our species^6–12^. Although rainforest ecosystems vary substantially across regions and should not be treated as direct analogues to each other, multidisciplinary investigations of four cave sites in Sri Lanka (Balangoda Kuragala, Batadomba-lena, Fa-Hien Lena and Kitulgala Beli-lena; Fig. 1) have provided an exceptionally rich and early archaeological record, ideal for understanding the earliest human adaptations to tropical environments and human tropical forest interaction over a long period^10,13–15^. These archaeological sites in the island’s lowland Wet and Intermediate Zones (Fig. 1) comprise a discontinuous record of human occupation reaching back to ~48,000 years ago (ka), providing some of the earliest direct evidence of human utilization of rainforest resources worldwide and several of the earliest *Homo sapiens* fossils found anywhere in South Asia^10,16,17^. Although chronological gaps exist at Fa-Hien Lena, probably reflecting local depositional or taphonomic processes rather than true absences of occupation^18^, the overall well-stratified cultural sequence of these cave and rockshelter sites, with consistent dating and no age reversals, points to sustained human presence in the region. Already in the oldest occupation layers, evidence suggestive of specialized hunting of arboreal and semi-arboreal small-bodied mammals can be found^10^, made possible through the use of microliths^13,15,19,20^, bone tools^11,21^ and what is currently recognized as the earliest bow-and-arrow technology documented outside of Africa^11^. Archaeological evidence further suggests that these populations gathered and processed freshwater and terrestrial molluscs, as well as fruits and nuts such as breadfruit and kekuna nut (also known as candlenut)^10^. This evidence contrasts with assumptions that coastal settings were preferred by humans migrating around the Indian Ocean rim^22–24^ and highlights our species’ ability to specialize, at the community level, in the use of more extreme terrestrial environments^25–28^.

Although Sri Lanka’s lowland Wet Zone and Intermediate Zone rainforest archaeological record is striking for its abundance of archaeological material in a tropical rainforest context, it has been challenging to glean reliable insights into the overall dietary reliance on specific types of resources, as zooarchaeological and archaeobotanical remains may represent isolated events (such as season-specific activities, hunting, butchering or food processing) or year-long occupation, constitute a time-averaged assemblage spanning an unknown period or offer skewed interpretations on the basis of the absence and presence of material within the archaeological record^29–31^. The application of stable carbon and oxygen isotope analysis to human fossils from these rainforest sites has revealed their relative reliance on closed forest or open habitat resources^9,14,18^. However, what these resources were (for example, plants versus animals versus freshwater fauna), and exactly how these populations utilized their ecosystem (that is, land use) over time, has remained somewhat obscure. This is particularly significant in Sri Lanka, where there is evidence for early connections between forest communities and communities living along the coast^11,13,32^, and palynological and phytolith evidence has been used to suggest early, intense relationships with plants in parts of the island that may even constitute cultivation akin to that seen in other tropical regions by the Early Holocene^33–35^. Human fossils from the lowland Wet Zone and Intermediate cave sites provide an unparalleled opportunity to study long-term dietary reliance on plants through time in Sri Lanka and to test hypotheses relating to plant and land use. To date, however, such work has been lacking owing to methodological limitations.

One major limitation has been the fact that dentine or bone collagen, widely used to assess the trophic level of a consumer with stable nitrogen isotope analysis (*δ*^15^N)^36^, rarely survives in tropical environments or in fossils older than ~20–100 ka because organic tissues degrade rapidly^37–39^. One way around this preservation problem is to turn to alternative, recently-developed independent proxies, such as zinc isotope ratios (*δ*^66^Zn)^12,40–46^ (see Supplementary Text 2 for an overview of zinc systematics in terrestrial ecosystems). Although zinc in the mineral phase of bone (and conversely dentine) is susceptible to diagenetic alteration^43,45^, tooth enamel’s dense and highly mineralized structure can effectively preserve biogenic zinc isotope signatures over archaeological and palaeontological timescales^45,47^ or under tropical conditions typically adverse to organic preservation^12,43,48^. Zinc stable isotope analysis has emerged as a dietary and trophic proxy capable also of separating omnivores from carnivores and herbivores^12,43,48^. Primarily controlled through the diet^49,50^, zinc isotope composition undergoes systematic mass-dependent fractionation across tissues and organs^49–54^, whereby soft tissues (including muscles) exhibit low values, leading to progressively lower *δ*^66^Zn values as trophic levels increase (−0.45‰ to −0.60‰, broadly comparable to the offset between muscle and diet^49,50^). Although zinc absorption varies among food sources, with animal products generally showing higher content^55,56^ and seemingly enhancing its absorption^57,58^, enamel *δ*^66^Zn consistently displays a clear herbivore–omnivore–carnivore structure (Supplementary Text 2) across comparable ecological settings. This novel method most notably suggested an omnivorous diet for one of the oldest modern human individuals found in Southeast Asia^12^ and unequivocally demonstrated a substantial plant-based component in the diets of hunter-gatherers from Taforalt, Morocco^46^. The latter challenges the prevailing notion of high dietary reliance on animals among pre-agricultural human groups and underscores the importance of investigating dietary practices before transitions to agriculture.

Here, we conduct a systematic comparison of *δ*^66^Zn values of tooth enamel from human specimens and a wide range of large mammals. We report novel *δ*^66^Zn data of 24 human and 57 mammal specimens from three Late Pleistocene–Holocene archaeological sites in Sri Lanka with established stratigraphic, chronological, archaeological and zooarchaeological frameworks (Fig. 1 and Supplementary Table 1), namely Balangoda Kuragala, Batadomba-lena and Fa-Hien Lena, for which *δ*^13^C and *δ*^18^O values measured on the same aliquots are already available^9,14,18^. As all sites lie within proximity to one another and share similar faunal communities, vegetation and geologies^9,14,18^, the dataset can be treated as a single regional record from the perspective of Zn analysis, and current understandings of Zn baseline behaviour in terrestrial ecosystems would suggest that no significant baseline differences should exist between sites or periods (Supplementary Text 2). Specifically, enamel *δ*^13^C values consistently indicate a C_3_-dominated rainforest food-web structure across sites and periods^9,14,18^, with only modest openings during the Last Glacial Maximum (LGM), and comparable tropical rainforest records suggest that such muted environmental variability does not generate substantial *δ*^66^Zn baseline shifts in the absence of major geological contrasts^12,43,48^. The sampled Sri Lanka fauna dataset is used to provide valuable information on *δ*^66^Zn ranges associated with different dietary behaviours and serve as a guideline for assessing dietary changes in human specimens. Human specimens from each of these three archaeological sites provide a record that, when combined, extends almost continuously across the last circa 20 ka, thus covering the end of the LGM (26.5 ka to 19 ka) (ref. ^59^), a major cold climatic event in the Last Glacial Cycle, up until the Holocene and reports of early plant cultivation in Sri Lanka and the earliest clear introduction of agricultural crops (that is millet) to the lowland Wet Zone of the island around 3 ka^19,35^. We aim to assess the changing relative reliance of hunter-gatherers from Sri Lanka on plants or animal matter across this key temporal period to better understand changing human–tropical forest relationships in this part of the world.

## Results

**Fig. 2:**
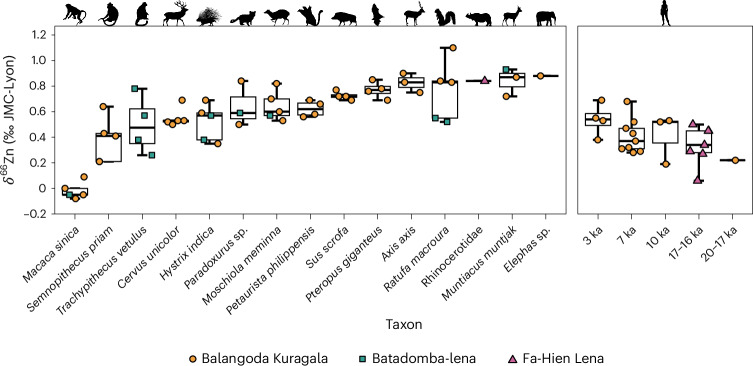
Box and whisker plots of the range of δ^66^Zn values (‰ JMC-Lyon) in tooth enamel for each taxon (*n* = 57) and between ages for the hunter–gatherer specimens (*n* = 24). Each colour and symbol represents specimens from different sites: Balangoda Kuragala (yellow circle, *n* = 61), Batadomba-Iena (turquoise square, *n* = 12) and Fa-Hien Lena (magenta triangle, *n* = 8). The boxes represent the 25th–75th percentiles, with the median represented by a bold horizontal line. The average analytical repeatability of *δ*^66^Zn measurements based on repeated analyses of the same sample (that is, standard deviation) was 0.03‰. For several taxa or age classes, sample sizes are *n* < 3 owing to the limited availability of suitable archaeological material. Silhouettes from PhyloPic under a Creative Commons license: *Macaca mulatta*, *Moschiola meminna*, *Petaurista leucogenys*, *Rhinoceros unicornis* and *Homo sapiens sapiens* (PDM 1.0); all other silhouettes available under a CC0 1.0 license.

All measured *δ*^66^Zn values from Balangoda Kuragala, Batadomba-lena and Fa-Hien Lena can be found in Fig. 2 and Supplementary Table 1, and all values from standard reference materials are reported in Supplementary Table 2.

The *δ*^66^Zn values (*n* = 81) obtained from tooth enamel range from 0.06‰ to 0.69‰ for humans (*n* = 24) and from −0.08‰ to 1.10‰ for the fauna (*n* = 57) (Fig. 2). The absence of a mixing line between zinc concentration and *δ*^66^Zn values (Supplementary Fig. 1) suggests no significant post-mortem zinc uptake, as further supported by Fourier transform infrared spectroscopy analyses previously conducted on these specimens^14^. Similarly, the comparison of *δ*^66^Zn values by tooth position, formation sequence and the full variability of later-forming teeth typically overlaps with that of early forming teeth, (Supplementary Fig. 2), indicating sampled enamel mineralization post weaning or, at the very least, that all *δ*^66^Zn values can be used equally. Finally, for species represented across multiple sites and time periods, *δ*^66^Zn values are homogeneous, thus indicating no meaningful spatial or temporal baseline differences and supporting the treatment of the faunal dataset as a single combined baseline. This mirrors *δ*^66^Zn data from Laos and Vietnam^12,43,48^, where sites spanning different climatic phases and vegetation histories nonetheless exhibit consistent baselines within taxa, thus demonstrating that regional climatic variation does not appear to significantly alter *δ*^66^Zn values in rainforest ecosystems.

## Discussion

Our zinc isotope dataset from Late Pleistocene to Late Holocene cave sites in Sri Lanka provides new, direct perspectives on long-term human dietary adaptations and patterns of land use within tropical rainforest environments.

Sri Lankan humans fall isotopically between herbivores and macaques, consistent with omnivory. Macaques play a central interpretive role: across Southeast Asia, they often show *δ*^66^Zn values overlapping with carnivores^12,43,48^, and in Sri Lanka, where obligate carnivores are absent, they occupy similar low *δ*^66^Zn ranks (Supplementary Text 2). Although their exact diet remains uncertain, their values are indistinguishable from carnivores elsewhere (Supplementary Text 2), and cross-site comparisons confirm that the *Δ*^66^Zn spacing between macaques and herbivores mirrors the herbivore–carnivore separation observed at other tropical sites (Supplementary Text 2). Modern *Macaca sinica* in Sri Lanka are known to devote a substantial proportion of their foraging time to acquiring nutritionally-important foods^60^ and have been observed foraging for eggs, insect larvae, lichens and, notably, small reptiles, mammals and birds^60,61^. Such dietary behaviour readily explains the low, carnivore-like *δ*^66^Zn values observed in the fossil record, as a trophic signal rich in animal-derived zinc would naturally result from this opportunistic feeding strategy. Moreover, although *δ*^66^Zn values appear to shift more linearly with diet composition^50^, unlike *δ*^15^N values that can appear ‘carnivorous’ with as little as ~20% animal protein^62–64^, animal tissues contain more zinc^55,56^ and promote greater absorption than plant foods^57,58^. As such, even moderate amounts of animal products could exert a stronger influence on a consumer’s *δ*^66^Zn, and carnivore-like values may not necessarily imply a predominantly carnivorous diet. Nonetheless, even if macaques are excluded, the distribution and structure of the remaining fauna align closely with those of herbivores and omnivores in Southeast Asia (Supplementary Text 2), reinforcing the robustness of the Sri Lankan isotopic baseline and enabling the confident placement of human values within a regional framework.

**Fig. 3:**
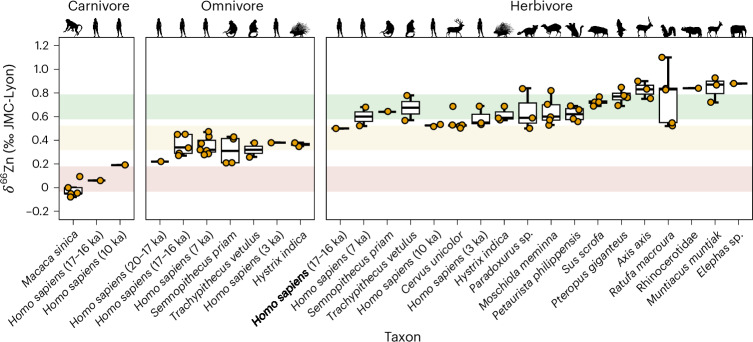
Dietary classification of the Sri Lankan fauna based on *δ*^66^Zn values. The box plots show *δ*^66^Zn distributions per taxon within each dietary facet (carnivore, omnivore and herbivore), with individual specimens plotted as points (*n* = 81). The dietary assignment of Sri Lankan specimens is based on kernel density estimation, which evaluates the posterior probabilities of class membership from *δ*^66^Zn values alone from previously published sites: Pà Hang Mountain, Tam Hay Marklot, Coc Muoi and Duoi U’Oi^12,43,48^. Coloured horizontal bands show 95% prediction intervals for site-level diet means derived from mainland Southeast Asian reference faunas: red for carnivores, yellow for omnivores and green for herbivores. These bands indicate where a typical site’s mean *δ*^66^Zn for each diet is expected to fall and are therefore narrower than the full spread of individual values. Together, the box plots and reference bands illustrate the placement of Sri Lankan taxa within trophic categories, while highlighting variability and classification uncertainty across groups. The boxes represent the 25th–75th percentiles, with the median represented by a bold horizontal line and whiskers indicating the minimum and maximum values. The taxa follow an ascending order per dietary group on the basis of their *δ*^66^Zn values. Silhouettes from PhyloPic under a Creative Commons license: *Macaca mulatta*, *Moschiola meminna*, *Petaurista leucogenys*, *Rhinoceros unicornis* and *Homo sapiens sapiens* (PDM 1.0); all other silhouettes available under a CC0 1.0 license.

To further test dietary interpretations, we applied a kernel density classification framework on the basis of *δ*^66^Zn values from well-characterized mainland Southeast Asian assemblages (Fig. 3; and Supplementary Text 2). As these comparative sites share similar rainforest food web structures (reflected in consistent internal *δ*^66^Zn ranges, isotopic spacing between trophic extremes and the shape of rank-order distributions; Supplementary Text 2) reference distributions can be confidently transferred to the Sri Lankan material. Posterior dietary assignments generated by this method corroborate the interpretations outlined above: *M. sinica* is consistently placed in the carnivore category, reinforcing its role as a trophic analogue for carnivores elsewhere despite its omnivorous ecology^60,61^. *Semnopithecus priam* and *Trachypithecus vetulus* are predominantly classified as omnivores, with several individuals falling into the herbivore category, in line with their mixed folivorous–frugivorous feeding strategies^65,66^. The predominance of omnivorous classifications in these colobines is somewhat unexpected, though this pattern may partly reflect taxonomic uncertainty between morphologically similar species, differences in the sampled tooth positions or developmental timing that capture distinct seasonal feeding phases or individual dietary flexibility linked to local ecological conditions. Future research focused on the long-term ecology and dietary behaviour of Sri Lanka’s extant colobines would be highly valuable for contextualizing both past and present dietary trends, particularly given the substantial anthropogenic disturbance that now shapes modern foraging patterns^67^. The same classification framework determined that humans (*n* = 24) are mostly designated as omnivores (*n* = 14), with two individuals assigned to the carnivore category and eight to the herbivore category, most of the latter belonging to younger phases of occupation. These assignments provide a probabilistic check on the relative placement of taxa within the Sri Lankan food web, highlighting both the robustness of cross-site trophic structure and the nuanced ecological variability of human diets.

Previous reconstructions of rainforest diets in Sri Lanka have been shaped largely by the preservation of faunal remains and durable hunting technologies (that is, stone and bone)^10,11,15,19–21,68^, which tend to bias interpretations towards meat consumption. As plant foods rarely survive in tropical archaeological contexts^69,70^, their contribution to subsistence has remained poorly assessed. The zinc isotope evidence helps correct this tendency, revealing that plant intake was a consistent and significant dietary component alongside animal resources. Moreover, the very low *δ*^66^Zn values of macaques in Sri Lanka, even though their diets almost certainly included a substantial proportion of plant foods^60,71,72^, highlight that omnivorous diets can isotopically resemble carnivory. This pattern could reflect the proportionally high zinc concentrations of animal tissues compared with plant resources^56^, which could skew isotopic mass balance even when plant matter is regularly consumed. The intermediate *δ*^66^Zn values observed for humans thus strongly suggest that the relative contribution of plant resources to their diets must have been considerable, since only a sustained intake of plant foods could counterbalance the isotopic signal of animal protein. This interpretation of a mixed diet aligns with archaeological evidence from Batadomba-lena, Fa-Hien Lena, Kebella-lena and Kitulgala Beli-lena, which indicates that both animal and plant resources were exploited: microliths, osseous tools and bow-and-arrow technology attest to the hunting of arboreal and semi-arboreal mammals, whereas molluscs, breadfruit and candlenut were also systematically collected and processed^10,11,15,19–21,68,73^. However, whereas the archaeological record, dominated by durable faunal remains and hunting implements, tends to emphasize animal consumption, the zinc isotope evidence suggests that plant resources perhaps contributed far more substantially to the diet than previously inferred. These findings are consistent with phytolith evidence from Fa-Hien Lena, which documents the presence of several wild plant taxa typical of today’s disturbed lowland rainforests, such as bananas, rice, breadfruit, durian, canarium and various palm and bamboo species^33^. Moreover, the diversity of wild banana phytoliths further could suggest multiple forms of exploitation beyond purely dietary^33^. As a result, when considered alongside the archaeological evidence and the established phytolith record, the zinc isotope data contribute to a more resolved, direct reconstruction of subsistence strategies, clarifying both the likely scope of plant resource use and its sustained role within human–rainforest interactions through time. A promising next step could be to pair enamel *δ*^66^Zn measurements with microremains (including phytoliths) recovered from dental calculus on newly excavated human remains, providing a rare opportunity to integrate an independent trophic proxy with direct, individual-level evidence of plant exploitation.

A key finding is the diachronic increase in *δ*^66^Zn values across our human sample. Linear regression shows a significant trend (Supplementary Fig. 3), with individuals from younger periods displaying higher *δ*^66^Zn values consistent with greater plant reliance. This indicates that Late Pleistocene foragers initially consumed proportionally more animal matter but progressively restructured their use of rainforest resources, incorporating a broader spectrum of plant foods through the terminal Pleistocene and Early Holocene. Although early suggestions of cultivation in highland Sri Lanka are based on changing vegetation cover in palaeoenvironmental cores, our data provide direct trophic evidence that plant resources became increasingly central to both diet and land use (that is, resource-use practices rather than land-cover change) in lowland rainforest hunter-gatherer populations through the Early and Middle Holocene. Importantly, this trajectory means that there was not a clear rupture with the arrival of the first clear domesticated crops in this part of Sri Lanka in the form of millet ~3 ka (refs. ^19,35^) but rather a deep-rooted adaptive shift already embedded in forager subsistence strategies. This long-term dietary shift complements previous stable isotope studies in Sri Lanka (see Supplementary Text 3 for an assessment of the relationship between *δ*^66^Zn and *δ*^13^C and *δ*^18^O values). Roberts et al.^9,14^ demonstrated heavy reliance on rainforest resources between ~36 and 3 ka, whereas Amano et al.^18^ emphasized continuity in rainforest occupation across climatic transitions. Yet, carbon and oxygen isotopes alone could not resolve trophic-level changes.

The zinc isotope data presented here fill this gap, revealing that the shift towards greater plant reliance began in the Late Pleistocene and continued through the Early–Middle Holocene, well before the proposed-emergence of crop-based agriculture in the lowlands around 3 ka (refs. ^9,14,35^), and may provide independent trophic context for debates about the earlier onset of cultivation activities in Sri Lanka^33–35^. The gradual increase in *δ*^66^Zn values through the Late Pleistocene and Holocene indicates a progressive reorganization of land use patterns, expressed here as increased reliance on plant procurement and processing, that probably facilitated the later incorporation of C_4_ crops into diets identified by Roberts et al. around 3 ka (ref. ^14^). Together, the multi-isotope evidence shows that the adoption of agriculture in Sri Lanka was not a sudden dietary break but the culmination of long-term adaptive processes within rainforest foraging economies. However, although the *δ*^66^Zn values in the current study provide trophic evidence for changing plant reliance, they do not quantify the proportion of cultivated foods in the diet, only their relative increase. Nonetheless, this integrated perspective helps reconcile the apparent stability of carbon and oxygen isotope baselines with the subtler yet significant trophic and behavioural shifts that redefined how tropical landscapes were exploited. The results reveal that humans consistently occupied an intermediate trophic position, between herbivores and higher trophic level, while also showing a significant diachronic increase in *δ*^66^Zn values. This trend indicates a persistent reliance on both plant and animal foods, coupled with a gradual but measurable shift towards increased plant consumption. However, future work assessing site-specific archaeological, geomorphological, botanical and faunal records across these rockshelters will be essential for refining our understanding of how long-term human settlement and rainforest use were shaped.

The Sri Lankan record also aligns with global evidence that plant reliance predates agriculture for which *δ*^66^Zn data is also available. At Taforalt in Morocco, the isotopic values indicate substantial plant intake in the Late Pleistocene^46^, overturning assumptions of animal-dominated diets among pre-agricultural groups. What sets Sri Lanka apart, however, is the diachronic resolution: although Taforalt provides a single snapshot of plant-focused foraging and probably niche construction, the Sri Lankan sequence demonstrates a gradual shift over tens of millennia. The intermediate trophic positioning in Sri Lanka is also mirrored at Tam Pà Ling, Laos, for a ~63 ka *H. sapiens* individual^12^, suggesting that mixed diets incorporating both animal and plant resources may have been a widespread and enduring feature of tropical rainforest adaptations in Southeast Asia. Notably, the value from the Tam Pà Ling *H. sapiens* individual falls in the lower range of values seen in Sri Lanka, associated with older periods. Although such direct comparison should be treated cautiously, given the different geographic and temporal contexts, the similarly low *δ*^66^Zn values from tropical rainforest *H. sapiens* help frame the broader context for interpreting dietary reconstruction in such settings. A comparison with Taforalt and Tam Pà Ling underscores the broader importance of zinc isotopes for capturing dietary trajectories rather than static states and highlights that intensification of plant use was a widespread but locally variable process.

Comparable developments across tropical Asia and Sahul reinforce this broader pattern of early and deliberate engagement with forest plant resources while recognizing that these records are not necessarily direct analogues to Sri Lanka in terms of ecology, chronology or cultural setting. At the Niah Caves in Borneo, human activity dating to between ~46 and 34 ka provides some of the earliest evidence for complex rainforest subsistence behaviours, including the detoxification of tropical plants and probable anthropogenic burning^7^. In the New Guinea highlands, archaeological deposits spanning ~49–36 ka document plant processing, alongside evidence for forest clearance and the transport of plant taxa across elevation zones^8^. Comparable strategies are evident across northern Sahul, from the coastal site of Kilu Cave (~33–29 ka) to the montane valleys of Ivane and Yuku (~49–15 ka), where macrobotanical remains and microfossils attest to the recurrent processing of root crops and nuts^74^. These findings reveal that tropical foragers across Asia and Oceania intensified plant exploitation and modified rainforest mosaics long before the advent of agriculture. Within this wider frame, the Sri Lankan zinc isotope trajectory, documenting a gradual rise in plant reliance between ~20 and 3 ka, thus provides direct trophic evidence for this process and is compatible with suggestions of earlier plant management or cultivation^33–35^, situating South Asia within a wider tropical pattern of pre-agricultural plant intensification and early landscape management^7,8,74^.

Our study further highlights the value of zinc isotope analysis in tropical archaeology, where collagen is often not preserved and *δ*^15^N-based trophic reconstruction is therefore frequently not feasible. Although carbon and oxygen isotopes are informative about habitat use, they do not provide direct trophic information. Zinc isotopes thus fill a crucial gap by providing an independent trophic proxy. The consistency of Δ^66^Zn spacing across Southeast Asian assemblages and the characteristic rank-order structure of food webs validate the robustness of this approach, even in contexts lacking carnivores. Limitations remain, notably sample sizes, the averaging effect of enamel and the atypically low, carnivore-like *δ*^66^Zn values observed in macaques, but the coherence of diachronic trends and alignment with archaeological records lend confidence to our interpretations. Samples for this study are derived from multiple Sri Lankan sites, and their integration into a single baseline should be interpreted cautiously, even if their proximity and shared ecological settings suggest broadly comparable isotopic conditions. Nevertheless, our zinc isotope record from Sri Lanka clearly demonstrates that humans occupied an intermediate trophic niche through time while gradually increasing their reliance on plant resources long before the advent of agriculture. This trajectory complements previous evidence for long-term rainforest occupation and highlights the adaptive flexibility of *H. sapiens* in tropical settings. Moreover, such a shift implies that humans were not simply hunting within forest faunal communities, as often emphasized in zooarchaeological records, but perhaps increasingly structuring their land use, without necessarily implying land-cover change, around the gathering and processing of diverse plant resources. In doing so, they point to a gradual reorientation of tropical forest resource use towards plant use long before the advent of agriculture. By providing the first diachronic trophic record of its kind, our study underscores the deep temporal roots of plant intensification and the role of rainforests not as barriers but as enduring habitats of opportunity for human societies.

## Methods

### Materials

Tooth enamel samples from humans (*n* = 24) and non-human mammals (*n* = 57) were analysed from three Late Pleistocene–Holocene archaeological sites in Sri Lanka: Balangoda Kuragala, Batadomba-lena and Fa-Hien Lena (Supplementary Table 1). Only specimens from well-defined stratigraphic units with robust chronological control were included (site-level Bayesian age modelling indicates coherent sequences without age reversals^19^), and taxonomic identifications follow established zooarchaeological practice as reported in the primary site publications, ensuring that the isotopic dataset is tied both to securely dated stratigraphic contexts and consistently identified faunal material.

A relatively low sample amount was used (average of 6.3 ± 2.6 mg), and no new sampling was undertaken. Instead, residual enamel powders from previous studies^9,14,18^ were analysed, ensuring no further semi-invasive sampling of these rare specimens and avoiding additional handling and transport of the archaeological material. Specimen selection was thus constrained by material availability: all available human specimens were included and, for non-human mammals, we prioritized taxa with broader representation (targeting *n* ≈ 5 where possible) and, when available, included specimens from multiple sites to evaluate whether site-level baseline differences were likely. Human samples are treated as tooth specimens because they derive from isolated dental remains, and the present analyses were performed on residual enamel powders from these teeth. Although each tooth could represent a different individual, this cannot be verified from isolated teeth alone. Accordingly, we use ‘specimen’ throughout and avoid individual-level claims.

Batadomba-lena (6°46′ N, 80°12′ E) is a small rockshelter situated near a stream in the rainforest foothills of Sri Pada, Ratnapura District. Excavations have revealed a long cultural sequence dated between ~36,000 and 12,000 calibrated (cal.) years BP, containing microliths, bone tools, faunal remains and human fossils, documenting repeated Late Pleistocene occupation within rainforest environments^14^. Fa-Hien Lena (6°36′ N, 80°13′ E; ~180 m above sea level), located ~30 km from the present coastline in Kalutara District, preserves several of the earliest known *H. sapiens* fossils in South Asia. Excavations have identified undisturbed Late Pleistocene deposits dated between ~48,000 and 37,000 cal. years BP, overlain by rich occupational horizons extending through the Terminal Pleistocene and Holocene (~13,000–4,500 cal. years BP) containing abundant lithic, osseous and botanical remains^9–11,15,18^. Balangoda Kuragala (6°37′ N, 80°52′ E), positioned along the ecological transition between the Wet and Dry Zones, provides evidence for human occupation spanning ~15,000 to 6,000 cal. years BP. Excavations have yielded microlithic tools, shell beads and well-preserved human and faunal assemblages, illuminating forager adaptations at the Pleistocene–Holocene boundary^9^.

As the sites lie within the lowland rainforest regions (Wet and Intermediate Zones) and share similar geological substrates, faunal communities and vegetation types, the material is treated as a single regional assemblage. The dataset encompasses specimens spanning ~20 to 3 ka, covering the end of the LGM through to the early stages of plant cultivation and agriculture on the island.

### Zinc chemical purification and measurement

Samples (*n* = 81) and aliquots of NIST SRM 1400 bone ash reference material (*n* = 10) were digested in Savillex beakers with 1 ml of HCl 1.0 M, evaporated and then redissolved in 1 ml HBr 1.5 M. Following this, zinc chemical purification was achieved by ion-exchange column chromatography, using preconditioned 10 ml hydrophobic interaction columns on 1 ml AG-1×8 resin (100–200 dry mesh size, 106–180 μm wet bead size). A total of 2 ml of HBr 1.5 M was used for matrix residue elution, followed by 5 ml of HNO_3_ 0.3 M for the zinc elution into new Savillex beakers. Samples were then dried and redissolved in 1 ml HBr 1.5 M to undergo the procedure again to fully remove the phosphate matrix. Finally, samples were dried and redissolved in 1 ml HNO_3_ 0.3 M before measurements. A procedural blank was also included and prepared alongside every chemical purification.

Zinc isotopic ratios measurement were performed on a Thermo Scientific Neptune Plus Multi-collector inductively coupled plasma mass spectrometry at the Federal Institute for Materials Research and Testing (Bundesanstalt für Materialforschung und-prüfung, Berlin, Germany), using the Cu-doping protocol of Toutain et al.^75^. Zinc isotope values are presented as relative isotope ratios in the delta (*δ*) notation:

$$\left.{{\delta }_{\mathrm{JMC-Lyon}}}^{66/64}({\mathrm{Zn}})=\left({66\atop}{\mathrm{Zn}}/{64\atop}{\mathrm{Zn}}_{\mathrm{smp}}\right)/\left({66\atop}{\mathrm{Zn}}/{64\atop}{\mathrm{Zn}}_{\mathrm{JMC}-{\mathrm{Lyon}}}\right)-1\right)$$

Samples have been bracketed with the Alfa Aesar in-house material and have been scale converted using equation 4 in Vogl and collaborators^76^ to the JMC-Lyon scale using an offset value of +0.27‰ for *δ*^66^Zn between JMC-Lyon and the in-house Alfa Aesar bracketing material^40,77^. Samples and standards were diluted to 100 µg g^−1^ for the measurements.

Reference material NIST SRM 1400 (bone ash) was prepared and analysed with the samples and had *δ*^66^Zn values of 0.92 ± 0.04‰ (*n* = 10) (Supplementary Table 2), in line with reported values^12,41–46,48–50,78,79^. Each sample and reference material aliquot were ideally measured two to three times, and the average standard deviation between measurements was 0.03 ± 0.03‰ (1*σ*, *n* = 81) and 0.03 ± 0.02‰ (1*σ*, *n* = 10), respectively. This level of repeatability between samples and international reference material demonstrates the high robustness of sample purification and analytical precision of the measurements. The average zinc content from the chemistry blanks was 5.7 ± 11.5 μg (1*σ*, *n* = 8).

### Statistical analyses

All statistical analyses were conducted using the open-source programme R software (R version 4.5.1^80^) using an alpha level for significance (that is, *P* values) of 0.05. Preliminary tests and inspection of the data were conducted when necessary to ensure the dataset fulfilled specific tests’ assumptions (for example, normally distributed and homogeneous residuals). All *R*^2^ and *P* values were adjusted using multi-testing correction when needed. The following R packages were used in the current study: ggplot2^81^, tidyverse^82^, cowplot^83^ and rstatix^84^.

### Reporting summary

Further information on research design is available in the Nature Portfolio Reporting Summary linked to this article.

## Supplementary information

Supplementary Information Supplementary Figs. 1–3, Tables 1–2, Text 1—zinc isotope systematics in terrestrial ecosystems, Text 2—interpreting trophic position in Sri Lankan food web through *δ*^66^Zn isotopic evidence, also including Figs. 4–8, Text 3—relationship between *δ*^66^Zn and *δ*^13^C/*δ*^18^O in human enamel, including Table 3.

Reporting summary

## Data Availability

All data generated or analysed during this study are included within this Article and its Supplementary Information.
