# Supplementary material for: Pre-agricultural intensification of plant use in Pleistocene Sri Lankan rainforests
Source: Nat Ecol Evol. 2026 May 20;10(7):1245–53. doi: 10.1038/s41559-026-03082-6 (PMC13345896; doi:10.1038/s41559-026-03082-6)
Supplement: Supplementary file 1 — Supplementary Figs. 1–3, Tables 1–2, Text 1—zinc isotope systematics in terrestrial ecosystems, Text 2—interpreting trophic position in Sri Lankan food web through δ66Zn isotopic evidence, also including Figs. 4–8, Text 3—relationship between δ66Zn and δ13C/δ18O in human enamel, including Table 3. [file 41559_2026_3082_MOESM1_ESM.pdf]

nature ecology & evolution

Supplementary information https://doi.org/10.1038/s41559-026-03082-6

Pre-agricultural intensification of plant use in Pleistocene Sri Lankan rainforests

In the format provided by the authors and unedited

Supplementary Information for:

Pre-Agricultural Intensification of Plant Use in Pleistocene Sri Lankan Rainforests

Nicolas Bourgon[1,2*], Marcus Oelze[2], Noel Amano[3], Oshan Wedage[4], Nimal Perera[5], Patrick Roberts[1]

1. Department of Coevolution of Land Use and Urbanisation, Max Planck Institute of Geoanthropology, Kahlaische Straße 10, D-07745 Jena, Germany
2. Bundesanstalt für Materialforschung und -prüfung (BAM), Richard-Willstätter-Straße 11, 12489, Berlin, Germany.
3. Max Planck Institute of Geoanthropology, Kahlaische Straße 10, D-07745 Jena, Germany
4. Department of History and Archaeology, University of Sri Jayewardenepura, Gangodawila, Nugegoda, Sri Lanka.
5. Excavation Branch, Department of Archaeology of the Government of Sri Lanka, Sir Marcus Fernando Mawatha street, 00700 Colombo, Sri Lanka.

**Corresponding author: bourgon@gea.mpg.de*

This file contains:

Supplementary Material – Supplementary Figures 1 – 3

Supplementary Material – Supplementary Tables 1 – 2

Supplementary Material – Text 1: Zinc isotope systematics in terrestrial ecosystems

Supplementary Material – Text 2: Interpreting trophic position in Sri Lankan foodweb through δ^{66}Zn isotopic evidence, and also including Supplementary Figures 4-8

Supplementary Material – Text 3: Relationship between δ^{66}Zn and δ^{13}C/δ^{18}O in human enamel, , and also including Supplementary Table 3

Supplementary Material – Figures

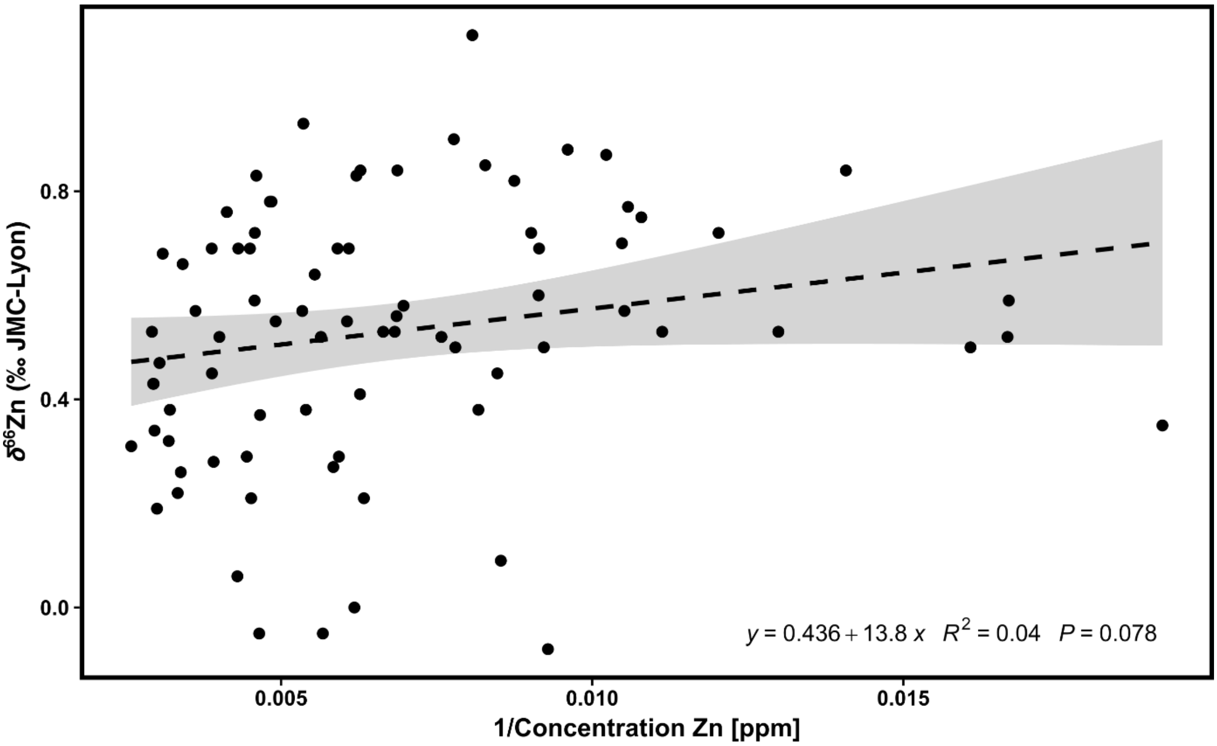

Supplementary Figure 1 | Relationship between δ^{66}Zn values and inverse Zn concentrations. Relationship between δ^{66}Zn values and the inverse of Zn concentration (1/Zn, ppm) for all specimens (n = 81). The fitted trendline with 95% confidence band is used to evaluate potential diagenetic alteration: a strong correlation between δ^{66}Zn and increasing/decreasing Zn concentration would indicate post-depositional exchange or leaching. In this dataset, the absence of a significant trend (p > 0.05) supports the geochemical integrity of the measured enamel values, as highlighted by already published FTIR results [1].

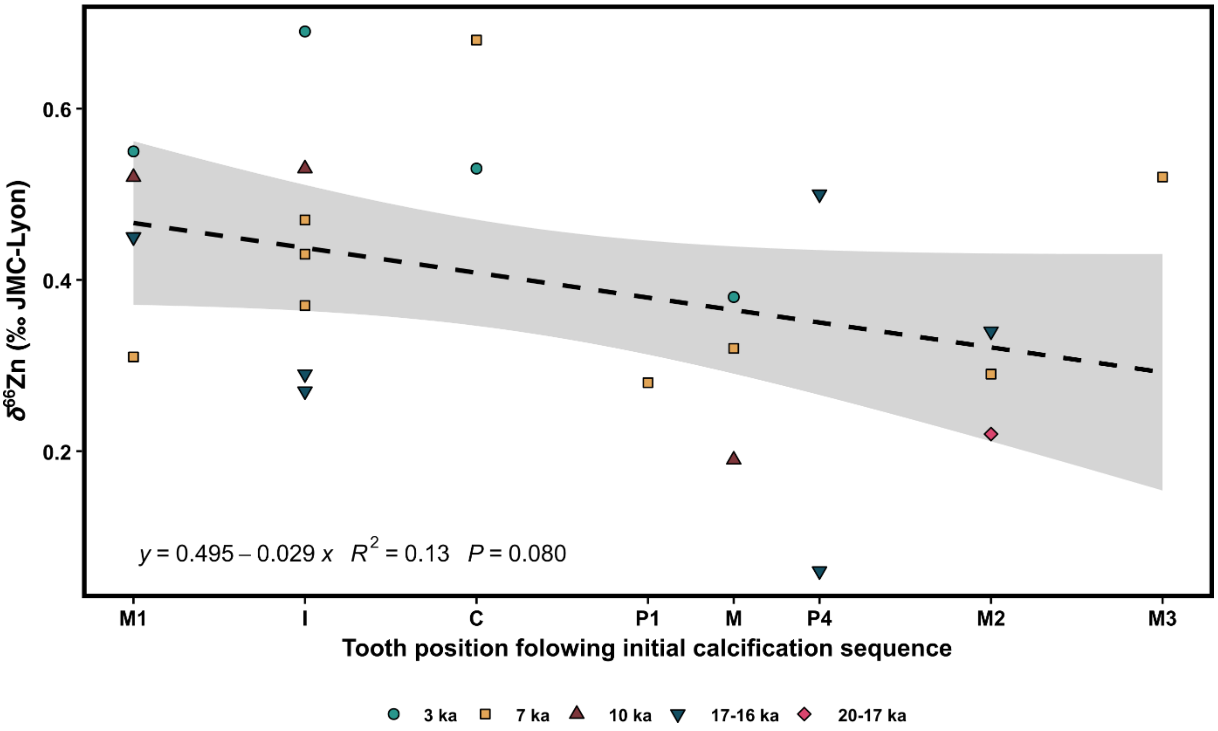

Supplementary Figure 2 | δ^{66}Zn values by tooth position in humans across developmental sequence. Relationship between δ^{66}Zn values and tooth position in humans (n = 24), ordered according to the sequence of calcification [2,3]. Individual data points are colored and shaped by chronological group, and the fitted trendline with 95% confidence band illustrates overall variation. In cases where individual molar position could not be determined (e.g., identified only as 'M' rather than 'M1', 'M2', or 'M3'), an averaged tooth-formation age derived from M1–M3 was used to represent their relative developmental position on the x-axis. No significant relationship is observed (p > 0.05), indicating that δ^{66}Zn values do not systematically differ between teeth forming in utero, during breastfeeding and weaning, or in later developmental stages. At minimum, substantial overlap in δ^{66}Zn values across tooth positions suggests continuity between early-life and adult dietary signatures.

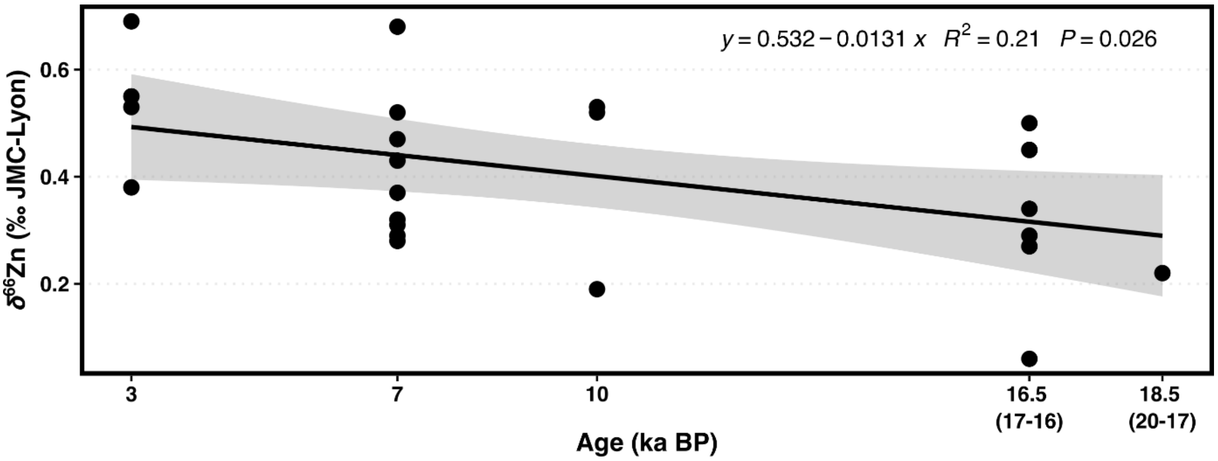

Supplementary Figure 3 | Temporal trend in human δ^{66}Zn values across the Late Pleistocene and Holocene. Relationship between δ^{66}Zn values and specimen age (ka) in humans (n = 24). For statistical analysis, midpoints were used for ranges (e.g., 17–16 ka as 16.5 ka; 20–17 ka as 18.5 ka). The fitted regression line with 95% confidence band shows a significant negative relationship, indicating that individuals from older periods typically exhibit lower δ^{66}Zn values. This temporal increase suggests a long-term shift in dietary composition, with younger populations relying more heavily on plant resources relative to earlier groups.

Supplementary Material – Tables

Sample ID	Site	Dating (ka)	Taxon	Element	$\delta^{66}Zn$	1 σ	[Zn] (ppm)
BK 130	Balangoda Kuragala	15-10	*Elephas* sp.	m	0.88	0.02	104
BK 6	Balangoda Kuragala	10	*Semnopithecus priam*	Rm3	0.41	0.12	160
BK 7	Balangoda Kuragala	10	*Semnopithecus priam*	Rm2	0.21	0.04	158
BK 57	Balangoda Kuragala	12	*Semnopithecus priam*	Rm2	0.43	0.03	340
BK 59	Balangoda Kuragala	12	*Semnopithecus priam*	Lp1	0.21	0.02	221
BK 64	Balangoda Kuragala	12	*Semnopithecus priam*	Li1	0.64	0.01	180
BK 74	Balangoda Kuragala	12	*Ratufa macroura*	i	0.84	0.02	159
BK 106	Balangoda Kuragala	7	*Ratufa macroura*	I	0.83	0.03	161
BK 116	Balangoda Kuragala	10	*Ratufa macroura*	I	1.10	0.02	124
BDL 44	Batadomba-lena	16-13	*Ratufa macroura*	Lm3	0.52	0.02	132
BDL 14	Batadomba-lena	16-13	*Ratufa macroura*	RM3	0.55	0.01	165
BKH 1	Balangoda Kuragala	10	*Homo sapiens*	Lm1	0.52	0.01	249
BKH 2	Balangoda Kuragala	7	*Homo sapiens*	Li	0.43	0.00	339
BKH 3	Balangoda Kuragala	3	*Homo sapiens*	Ri	0.69	0.03	257
BKH 4	Balangoda Kuragala	7	*Homo sapiens*	RI	0.47	0.03	328
BKH 5	Balangoda Kuragala	7	*Homo sapiens*	RM1	0.31	0.03	386
BKH 7	Balangoda Kuragala	10	*Homo sapiens*	Lm	0.19	0.04	333
BKH 9	Balangoda Kuragala	7	*Homo sapiens*	RM2	0.29	0.02	225
BKH 10	Balangoda Kuragala	3	*Homo sapiens*	LC	0.53	0.02	342
BKH 11	Balangoda Kuragala	3	*Homo sapiens*	m	0.38	0.02	311
BKH 12	Balangoda Kuragala	7	*Homo sapiens*	m	0.32	0.01	313
BKH 13	Balangoda Kuragala	7	*Homo sapiens*	LP1	0.28	0.02	255
BKH 14	Balangoda Kuragala	7	*Homo sapiens*	Li	0.37	0.06	215
BKH 15	Balangoda Kuragala	10	*Homo sapiens*	RI	0.53	0.03	151
BKH 16	Balangoda Kuragala	7	*Homo sapiens*	LC	0.68	0.01	323
BKH 17	Balangoda Kuragala	3	*Homo sapiens*	RM1	0.55	0.04	204
BKH 19	Balangoda Kuragala	20-17	*Homo sapiens*	RM2	0.22	0.01	300
BKH 21	Balangoda Kuragala	7	*Homo sapiens*	Rm3	0.52	0.06	177
BYP209	Fa-Hien Lena	17-16	*Homo sapiens*	p4	0.50	0.05	128
BYP210	Fa-Hien Lena	17-16	*Homo sapiens*	i1	0.29	0.02	169
BYP212	Fa-Hien Lena	17-16	*Homo sapiens*	i	0.27	0.03	171
BYP213	Fa-Hien Lena	17-16	*Homo sapiens*	m1	0.45	0.02	118
BYP214	Fa-Hien Lena	17-16	*Homo sapiens*	P4	0.06	0.04	233
BYP215-1	Fa-Hien Lena	17-16	*Homo sapiens*	M2	0.34	0.05	337
BYP215-2	Fa-Hien Lena	17-16	*Homo sapiens*	M1	0.45	0.11	257
BDL 17	Batadomba-lena	19-16	*Hystrix indica*	p	0.57	0.01	95
BK 8	Balangoda Kuragala	10	*Hystrix indica*	M	0.59	0.00	60
BK 69	Balangoda Kuragala	15-10	*Hystrix indica*	Rp1	0.35	0.05	52
BK 91	Balangoda Kuragala	12	*Hystrix indica*	Lp	0.69	0.02	232
BDL 24	Batadomba-lena	16-13	*Hystrix indica*	LM2	0.38	0.01	122
BK 58	Balangoda Kuragala	6	*Pteropus giganteus*	m	0.85	0.01	121

BK 86	Balangoda Kuragala	7-6	*Pteropus giganteus*	m	0.69	0.02	222
BK 87	Balangoda Kuragala	12	*Pteropus giganteus*	m	0.76	0.04	242
BK 122	Balangoda Kuragala	12	*Pteropus giganteus*	m	0.78	0.03	206
BK 28	Balangoda Kuragala	7-6	*Petaurista philippensis*	M3	0.56	0.01	146
BK 50	Balangoda Kuragala	10	*Petaurista philippensis*	LM	0.58	0.01	144
BK 65	Balangoda Kuragala	7 ka	*Petaurista philippensis*	Rm3	0.69	0.01	164
BK 125	Balangoda Kuragala	7 ka	*Petaurista philippensis*	M	0.66	0.02	293
BK 34	Balangoda Kuragala	12 ka	*Muntiacus muntjak*	M	0.87	0.01	98
BK 78	Balangoda Kuragala	3 ka	*Muntiacus muntjak*	Lm2	0.72	0.04	218
BDL 28	Batadomba-lena	16-13	*Muntiacus muntjak*	Lp1	0.93	0.03	187
BK 1	Balangoda Kuragala	3	*Axis axis*	Lp2	0.75	0.03	93
BK 29	Balangoda Kuragala	10	*Axis axis*	RM	0.90	0.01	129
BK 77	Balangoda Kuragala	7-6	*Axis axis*	Lp	0.83	0.01	217
BK 33	Balangoda Kuragala	12	*Paradoxurus* sp.	Lp2	0.50	0.02	108
BK 30	Balangoda Kuragala	10	*Paradoxurus* sp.	m2	0.84	0.01	146
BDL 27	Batadomba-lena	16-13	*Paradoxurus* sp.	Lm2	0.59	0.03	219
BDL 38	Batadomba-lena	15-10	*Trachypithecus vetulus*	Rc	0.38	0.02	185
BDL 39	Batadomba-lena	15-10	*Trachypithecus vetulus*	Lm1	0.78	0.03	207
BDL 11	Batadomba-lena	20-19	*Trachypithecus vetulus*	Lp2	0.26	0.08	295
BDL 9	Batadomba-lena	15-10	*Trachypithecus vetulus*	LM3	0.57	0.02	276
BYP208	Fa-Hien Lena	12-8	Rhinocerotidae	P4	0.84	0.04	71
BK 35	Balangoda Kuragala	15	*Cervus unicolor*	Rp2	0.53	0.24	77
BK 37	Balangoda Kuragala	10	*Cervus unicolor*	Lp1	0.52	0.01	60
BK 84	Balangoda Kuragala	15-10	*Cervus unicolor*	Rm2	0.53	0.02	90
BK 102	Balangoda Kuragala	15-10	*Cervus unicolor*	RP1	0.50	0.01	62
BK 94	Balangoda Kuragala	10	*Cervus unicolor*	m	0.69	0.02	169
BK 90	Balangoda Kuragala	7-6	*Moschiola meminna*	Rm	0.82	0.03	114
BK 36	Balangoda Kuragala	6	*Moschiola meminna*	Rm3	0.70	0.01	95
BK 71	Balangoda Kuragala	12	*Moschiola meminna*	m3	0.60	0.02	109
BK 75	Balangoda Kuragala	10	*Moschiola meminna*	Rm3	0.53	0.03	146
BDL 23	Batadomba-lena	16-13	*Moschiola meminna*	Lm3	0.57	0.02	187
BK 9	Balangoda Kuragala	15-10	*Macaca sinica*	LM2	0.09	0.05	117
BK 20	Balangoda Kuragala	10	*Macaca sinica*	Rm3	0.00	0.10	162
BDL 1	Batadomba-lena	15-10	*Macaca sinica*	RM3	-0.05	0.04	176
BK 16	Balangoda Kuragala	10	*Macaca sinica*	Rm3	-0.05	0.02	215
BK 31	Balangoda Kuragala	10	*Macaca sinica*	Rm3	-0.08	0.06	108
BK 97	Balangoda Kuragala	7	*Sus scrofa*	Rm3	0.77	0.01	95
BK 121	Balangoda Kuragala	7-6	*Sus scrofa*	RC	0.72	0.01	83
BK 129	Balangoda Kuragala	7-6	*Sus scrofa*	Rc	0.72	0.01	111
BK 2	Balangoda Kuragala	10	*Sus scrofa*	LI1	0.69	0.01	109

Supplementary Table 1 | Sample material and isotope results. Sample metadata and isotopic results, including sample ID, site, dating (in ka), taxon, tooth position (element), δ^{66}Zn (‰ JMC-Lyon), δ^{66}Zn standard deviation (1σ), and zinc concentration (ppm). Dating follows previously published sources [1,4,5]. For tooth position, lowercase and uppercase letters for teeth indicate lower and upper teeth, respectively, while "R" indicates "right" and "L" indicates "left". Lowercase letters are also used where upper or lower assignment could not be determined.

ID	$\delta^{66}Zn$ (‰ JMC-Lyon)	1 σ
NIST SRM-1400 79	0.85	0.01
NIST SRM-1400 80	0.93	0.03
NIST SRM-1400 81	0.88	0.02
NIST SRM-1400 85	0.89	0.03
NIST SRM-1400 86	0.94	0.03
NIST SRM-1400 87	0.96	0.01
NIST SRM-1400 88	1.01	0.03
NIST SRM-1400 92	0.93	0.05
NIST SRM-1400 94	0.93	0.07
NIST SRM-1400 95	0.92	0.02

Supplementary Table 2 | Standard reference material isotope results. Isotopic results for the bone ash standard reference material NIST SRM-1400, including sample ID, $\delta^{66}Zn$ (‰ JMC-Lyon), and $\delta^{66}Zn$ standard deviation (1σ).

Supplementary Material – Text 1: Zinc isotope systematics in terrestrial ecosystems

Stable zinc isotope analysis is a relatively recent approach in ecological, archaeological and palaeontological research [6–16]. Zinc has five stable isotopes (^{64}Zn, ^{66}Zn, ^{67}Zn, ^{68}Zn, ^{70}Zn), with $\delta^{66}Zn$ (reported relative to $^{66}Zn/^{64}Zn$) being the most widely reported ratio in ecological and dietary applications. The majority of zinc isotope applications have focused on bioapatite tissues, particularly tooth enamel. Multiple studies indicate that enamel (and enameloid) can retain biogenic $\delta^{66}Zn$ values over long timescales, including in tropical environments and across geological timeframes, whereas Zn in bone mineral (and conversely dentine) is more susceptible to diagenetic alteration [8,9,12,17].

$\delta^{66}Zn$ analysis has emerged as an independent trophic proxy, notably permitting separation of omnivores from carnivores and herbivores [9–11]. In animals, Zn isotope composition is primarily diet-derived and undergoes mass-dependent fractionation within the body and along food chains [18,19]. Soft tissues such as muscle exhibit comparatively low $\delta^{66}Zn$ values, and because muscle is a dominant component of prey tissues consumed by most predators, consumers' $\delta^{66}Zn$ values tend to decrease with increasing trophic level in a broadly predictable manner [18–22]. Reported trophic spacing is commonly on the order of ~0.45–0.60‰ in some terrestrial food webs, consistent with diet–tissue offsets observed between muscle and diet [18,19], though the exact magnitude can still vary with feeding ecology, such as the relative contribution of plant, skeletal, and animal soft tissues in carnivore diets [7,9–11].

Dietary zinc intake and the fraction absorbed depend not only on the zinc content of ingested resources but also on intestinal bioaccessibility and bioavailability, which themselves can be influenced by certain food components [23]. Phytate, abundant in some plant foods, can markedly reduce zinc bioavailability [24,25], such that plant-based diets often provide less bioavailable Zn than animal-based diets [26,27]. In contrast, dietary proteins are generally associated with higher zinc uptake [28,29], particularly animal proteins [30], and adding animal protein to plant-based diets can substantially improve zinc bioavailability, likely by counteracting phytate inhibition [31,32]. Moreover, many animal products are zinc-rich [33,34], whereas most commonly consumed plants are lower in zinc, with cereals, seeds, and nuts being notable exceptions, though these are also typically phytate-rich, which can strongly constrain absorption [33–35]. In principle, these factors related to zinc absorption could bias $\delta^{66}Zn$ values toward animal-derived resources in mixed diets. However, available evidence from terrestrial food webs [9–11] and controlled experiments [19] suggests that such effects do not overwhelm the trophic structure recorded by enamel $\delta^{66}Zn$. Taxa with omnivorous opportunistic feeding ecologies typically show intermediate values between herbivores and carnivores, rather than falling into the carnivore range [9–11]. Accordingly, $\delta^{66}Zn$ distributions commonly display a three-mode structure (herbivore–

omnivore–carnivore), in contrast to the more binary separation observed for $\delta^{15}N$ [11]. In turn, this distinction between these two trophic proxies supports the interpretation of omnivory as a distinct isotopic position rather than a methodological or classification issue. Taken together, zinc bioavailability and absorption likely contribute to inter-individual variability and mass-balance effects, but they currently do not appear to obscure the dominant dietary signal at the level of broad trophic category.

Across terrestrial food webs, variability in zinc stable isotope baselines is derived from soils and the zinc accessible to plants. Geological substrates, as well pedogenic and biogeochemical processes that partition Zn among mineral and organic fractions, are the primary factors that shape $\delta^{66}Zn$ values for given food webs [36–38]. In tropical soils in particular, $\delta^{66}Zn$ signatures can differ systematically from non-tropical settings because intense weathering, leaching, and secondary mineral formation alter the composition and speciation of Zn [37]. The synthesis by Liang et al. highlights that precipitation of secondary Fe oxides and organic complexation can preferentially retain lighter Zn isotopes in highly weathered tropical soils, while extreme leaching can export substantial Zn, often contributing to Zn deficiency in tropical regions, and influence dissolved/riverine $\delta^{66}Zn$ toward heavier values relative to crustal averages at short timescales and regional scales [37,39,40].

Plants fractionate Zn isotopes during uptake and internal transport, with transport dynamics being the primary influence over $\delta^{66}Zn$ values across plants organs and plants species [41–43]. As a result, leaves often exhibit lower $\delta^{66}Zn$ values than stems [39,44–48], and higher-growing taxa such as shrubs and trees may show lower $\delta^{66}Zn$ than lower-growing herbaceous plants and grasses [39,44,45], patterns that can, in some ecosystems, translate into grazer–browser differences at the consumer level [7,16,49].

More broadly, while spatial differences in faunal $\delta^{66}Zn$ baselines have been reported, the drivers of these differences remain an active area of research. Temporal shifts in faunal baseline $\delta^{66}Zn$ are also not yet well characterized, in part because the ecological and archaeological applications of Zn isotopes remain relatively young. Nonetheless, $\delta^{66}Zn$ baseline differences appear minimal when the underlying bedrock composition is comparable [9–11]. In both Laos and Vietnam, faunal assemblages spanning multiple sites and depositional ages exhibit consistent $\delta^{66}Zn$ values within taxa, with only those localities underlain by distinct lithologies showing measurable offsets [11]. Moreover, $\delta^{66}Zn$ values remain broadly stable even across climatic and environmental fluctuations documented by carbon and oxygen isotope records, thus reinforcing that short-term vegetation or hydrological changes exert limited influence on baseline Zn isotope compositions in tropical rainforest settings. In contrast, assemblages from open environments (e.g., savannahs [7], Pleistocene European cold steppes [15,16], and Iberian grasslands [49]) display markedly different baseline signatures. Together, these observations suggest that $\delta^{66}Zn$ baselines are governed primarily by broad, macro-scale soil and ecosystem

processes (e.g., rainforest versus grassland biogeochemical regimes), rather than by fine-scale, site-specific or temporal micro-environmental variations.

In the context of the current study, the three Sri Lankan sites included are all situated within Sri Lanka's lowland wet and intermediate zones and are separated by at most ~70 km. Although Zn isotope baselines can vary with soil mineralogy and biogeochemical cycling [36–38], available evidence from tropical rainforest settings in Laos and Vietnam indicates broadly comparable faunal δ^{66}Zn baselines across sites and climatic phases, with clear divergences primarily associated with contrasting bedrock/geologic substrates [11]. Stable carbon isotope evidence in Sri Lanka indicates a persistent overlapping C_3 rainforest signal across sites and ages [1,4,5], with low enamel δ^{13}C values close to or within the closed-canopy range. Consistent with the broader pattern of baseline differences between markedly different ecosystems (e.g., rainforest versus grassland), we therefore treat the assemblage as a single regional dataset for baseline framework and dietary interpretation.

Supplementary Material – Text 2: Interpreting trophic position in Sri Lankan foodweb through δ^{66}Zn isotopic evidence

The case of *Macaca sinica*

The interpretation of trophic structure in the Sri Lankan fossil foodweb, particularly the trophic position of macaques *(Macaca sinica* and *Macaca* sp.), benefits from an integrative cross-site analysis of δ^{66}Zn values spanning multiple assemblages across South and Southeast Asia. Several lines of isotopic and statistical evidence suggest that macaques in Sri Lanka occupied a higher trophic level than herbivorous or primarily frugivorous taxa, and in fact likely consumed a substantial proportion of animal matter. Below, we synthesize the evidence from comparative isotopic structure, bootstrapped means and confidence intervals, and rank-ordered foodweb distributions.

Across the five localities analysed (namely Sri Lanka, Pà Hang Mountain, Tam Hay Marklot, Coc Muoi, and Duoi U'Oi; [9–11]), the δ^{66}Zn values of the fossil communities show consistent internal ranges and distributions. All of these sites are located in tropical Southeast Asia and are characterized by broadly similar tropical rainforest environments [1,4,9,50,51]. While some ecological and taxonomic differences exist among them, the overall structure of their δ^{66}Zn isotopic spacing (Δ^{66}Zn) reflect a high degree of comparability. This coherence stands in marked contrast to other published assemblages from ecologically distinct regions such as Koobi Fora (Kenya), Ranis (Germany), Gabasa (Spain), Taforalt (Morocco), or Gironde (France), where environmental context, faunal structure, and trophic baselines differ substantially from the tropical forest ecosystems examined here [7,15,16,49]. In this context, comparing macaques' δ^{66}Zn values across sites (**Supplementary Supplementary Figure *4***) underscores a clear pattern, with individuals from Sri Lanka exhibiting consistently lower values than their counterparts elsewhere.

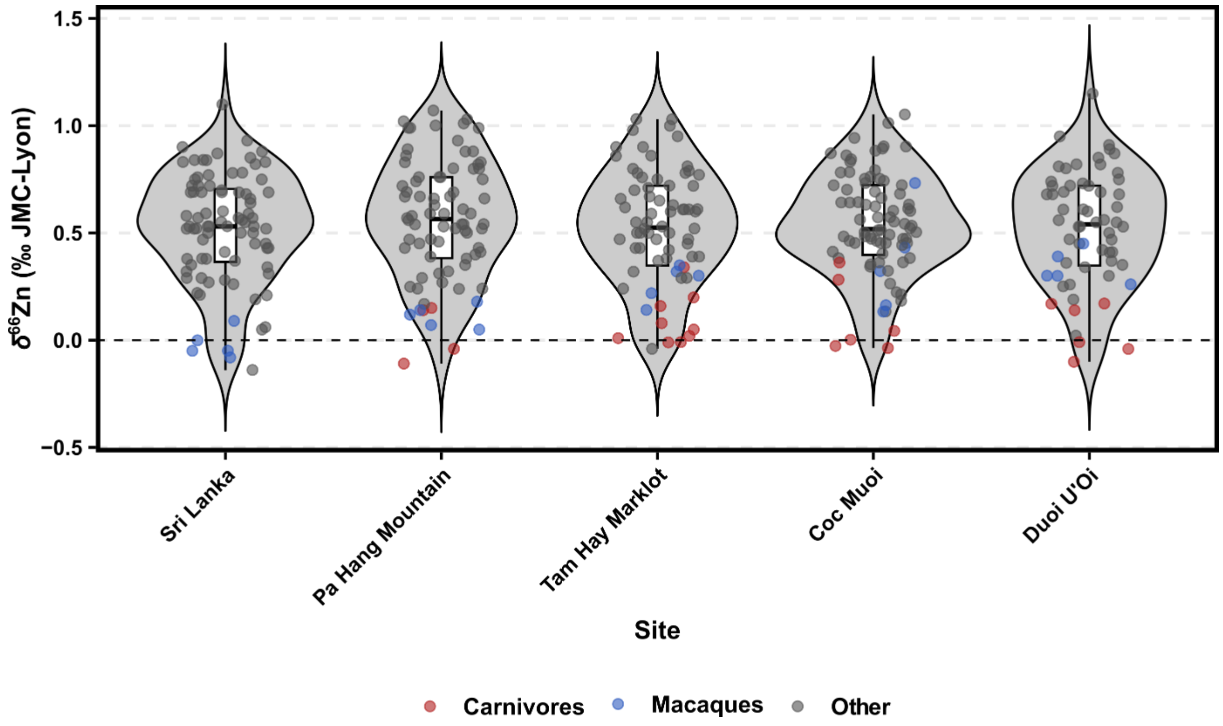

Supplementary Figure 4 | δ^{66}Zn distributions across five tropical Southeast Asian fossil assemblages. Violin plots of δ^{66}Zn values across five tropical Southeast Asian fossil assemblages (Sri Lanka, *n* = 81; Pà Hang Mountain, *n* = 76; Tam Hay Marklot, *n* = 72; Coc Muoi, *n* = 84; and Duoi U'Oi, *n* = 60). Each violin represents the full distribution of δ^{66}Zn values at a given site, with internal boxplots showing the interquartile range, median, minimum–maximum values. Macaque specimens are highlighted in blue, and carnivores in red. The data reveal broadly comparable δ^{66}Zn ranges and distributions across sites. Notably, macaque individuals from Sri Lanka exhibit systematically lower δ^{66}Zn values than their conspecifics elsewhere, falling within the range of carnivores from Laos and Vietnam, and consistent with the lowest macaques' values observed at other sites.

Notably, we also find that the isotopic spacing between the taxa with the lowest and highest δ^{66}Zn values ($\Delta^{66}Zn_{max\text{-}min}$, where Δ denotes the range in δ^{66}Zn values, here calculated as the difference between the maximum and minimum values) remains stable across sites in tropical Southeast Asia (**Supplementary Supplementary Figure *5***), typically separating herbivores (high values) from carnivores (low values). This baseline consistency validates cross-site comparisons and supports the use of δ^{66}Zn as a reliable trophic indicator across tropical forest environments. In Sri Lanka, however, where no unambiguous carnivores are present in the assemblage, the macaques emerge as the lowest-δ^{66}Zn taxon, even below all identified omnivores.

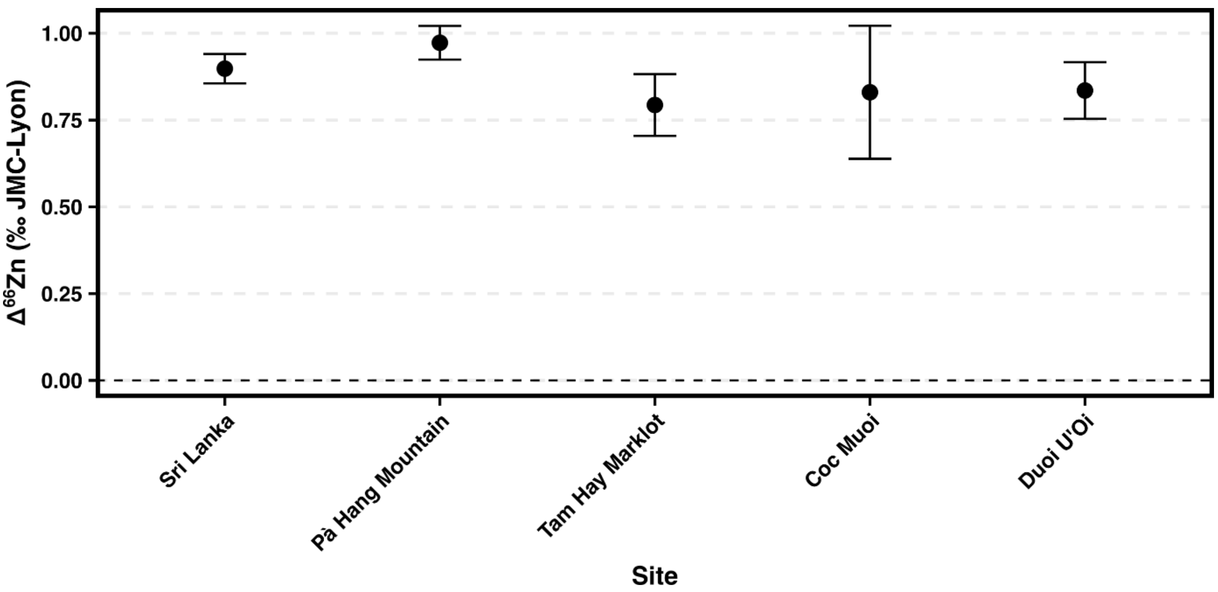

Supplementary Figure 5 | *Δ^{66}Zn spacing between highest and lowest trophic positions across five fossil assemblages.* Δ^{66}Zn values across five tropical Southeast Asian fossil sites (Sri Lanka, Pà Hang Mountain, Tam Hay Marklot, Coc Muoi, and Duoi U'Oi), calculated as the difference between the mean δ^{66}Zn values of the taxon with the highest values and the one with the lowest values at each site. Error bars represent propagated standard errors of the means (SE) between the highest and lowest taxa per site. As the taxa with the highest δ^{66}Zn values only had a single specimen for both Sri Lanka and Pa Hang Mountain, the measurement repeatability of ±0.03 ‰ was used as a minimum SE to allow error propagation. In most sites, the resulting Δ^{66}Zn value reflects the expected isotopic separation between herbivores and carnivores. In Sri Lankan, however, the lowest δ^{66}Zn values are observed in macaques, which replace carnivores in defining the lower end of the isotopic range. Despite the absence of obligate carnivores, the Δ^{66}Zn spacing at Sri Lanka closely matches that of the other sites, reinforcing the interpretation of macaques as occupying a comparably high trophic position within the local food web.

Importantly, the δ^{66}Zn values of Sri Lankan macaques are similar to those of known carnivores (e.g., tigers, leopards, and canids) in Laos and Vietnam, highlighting their potential consumption of high-trophic-level food sources (e.g., invertebrates, small vertebrates, eggs). Crucially, the magnitude of $\Delta^{66}Zn_{\text{Macaques-herbivores}}$ values in Sri Lanka is indistinguishable from that of $\Delta^{66}Zn_{\text{carnivores-herbivores}}$ at the other sites (**Supplementary Supplementary Figure *6***), strongly suggesting that macaques occupied a higher trophic position, at least isotopically (Z = −0.78, p = 0.44).

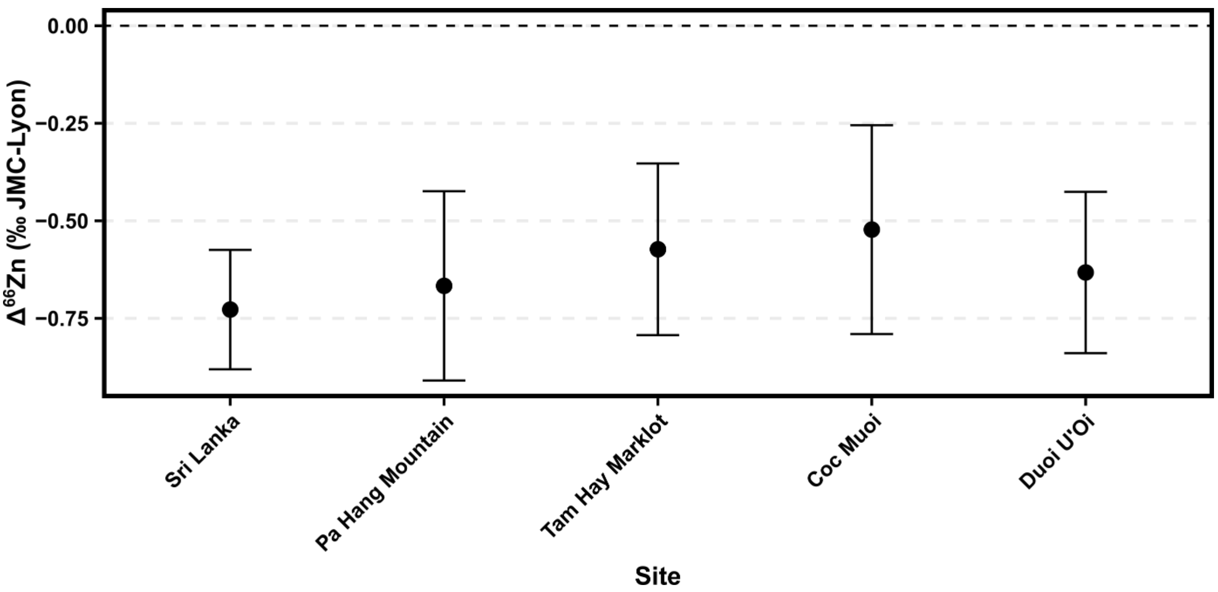

Supplementary Figure 6 | Consistent Δ^{66}Zn trophic spacing across five fossil assemblages. Δ^{66}Zn values across five tropical Southeast Asian fossil assemblages (Sri Lanka, Pà Hang Mountain, Tam Hay Marklot, Coc Muoi, and Duoi U'Oi), calculated as the difference between the mean δ^{66}Zn values of herbivores and carnivores at each site, and with error bars representing the propagated standard errors of the means (SE). At Sri Lanka, where obligate carnivores are absent, macaques are used as a proxy for the highest trophic position. The resulting Δ^{66}Zn spacing at Sri Lanka is nearly identical to that observed in sites with established herbivore–carnivore baselines, suggesting that macaques fulfil a similar position within the local trophic structure. This isotopic consistency reinforces the interpretation of macaques as a higher trophic taxon in the Sri Lankan context.

Bootstrapped means and confidence intervals further support this interpretation. Across all sites, macaques consistently display lower δ^{66}Zn values than both herbivores and omnivores, even when accounting for interspecific variance through bootstrapping (**Supplementary Supplementary Figure *7***). While omnivores and herbivores occasionally exhibit minor overlap, macaques often lies outside or below both groups, and completely outside in Sri Lanka, reinforcing its unique isotopic position within the foodweb. This statistical framing helps insulate the interpretation from sample size artifacts and inter-individual variability, something particularly important given the limited number of species in the Sri Lankan "pure" herbivore set.

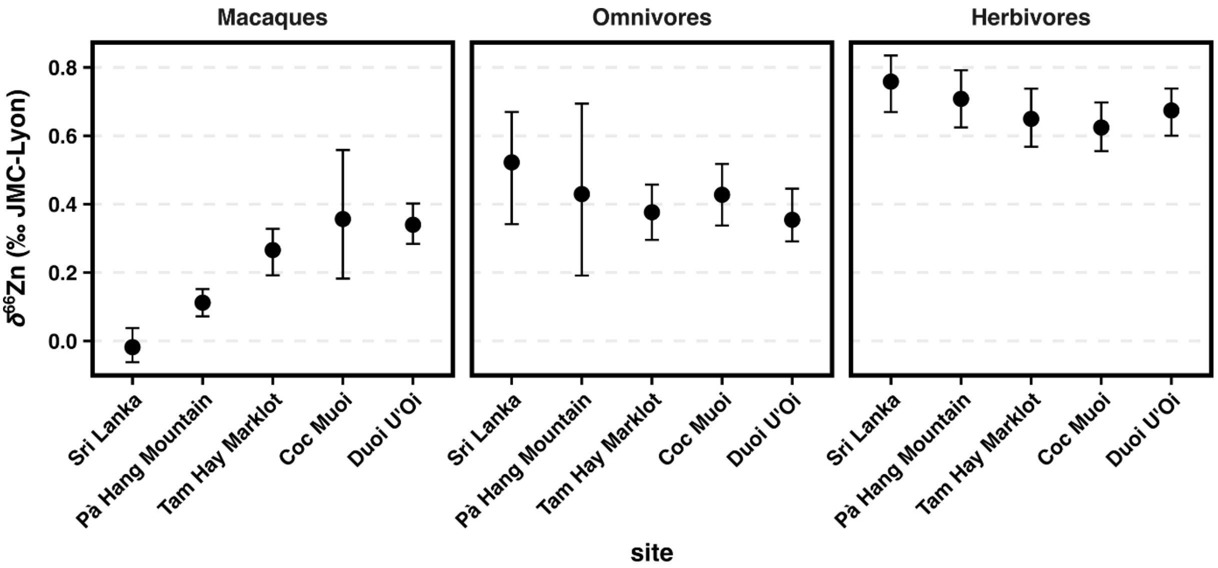

Supplementary Figure 7 | Bootstrapped δ^{66}Zn means and confidence intervals across macaques, dietary groups and fossil assemblages. Bootstrapped mean δ^{66}Zn values and 95% confidence intervals for macaques, omnivores, and herbivores across five tropical Southeast Asian fossil assemblages (Sri Lanka, Pà Hang Mountain, Tam Hay Marklot, Coc Muoi, and Duoi U'Oi). The macaques' values from Sri Lanka are consistently lower than all other dietary groups at any site. In most cases, confidence intervals for omnivores and herbivores show limited or no overlap, indicating distinct trophic isotopic signatures. While macaques occasionally overlap with omnivores at some sites, the Sri Lankan macaque's confidence interval lies clearly below all macaques, omnivores and herbivores, reinforcing its distinct isotopic profile and supporting its interpretation as a higher trophic level taxon within this assemblage.

Rank-order plots of δ^{66}Zn values per site provide a visual summary of internal trophic structure across sites. In the case of Sri Lanka, macaques occupy the lowest ranks (**Supplementary Supplementary Figure *8***), a position otherwise filled by carnivores at the other localities. This reinforces the idea that, isotopically speaking, macaques in Sri Lanka fulfils a higher trophic position, further suggesting dietary reliance on high-trophic-level items (invertebrates, small vertebrates, etc.). Beyond this ordering, the shape of the rank-order curves reveals a strikingly consistent pattern across sites. Typically, a steep slope appears at the lower end of the distribution (corresponding to carnivores or macaques in Sri Lanka), followed by a "shoulder" and a long gradual incline and ending in a second inflection near the uppermost values. This somewhat sigmoidal structure appears robust to differences in sample size and taxonomic composition, suggesting that it reflects a conserved trophic architecture across Southeast Asia's forest ecosystems. Such patterns imply not only consistent isotopic spacing between trophic levels but also similar proportions and ecological breadth of dietary niches within each food web.

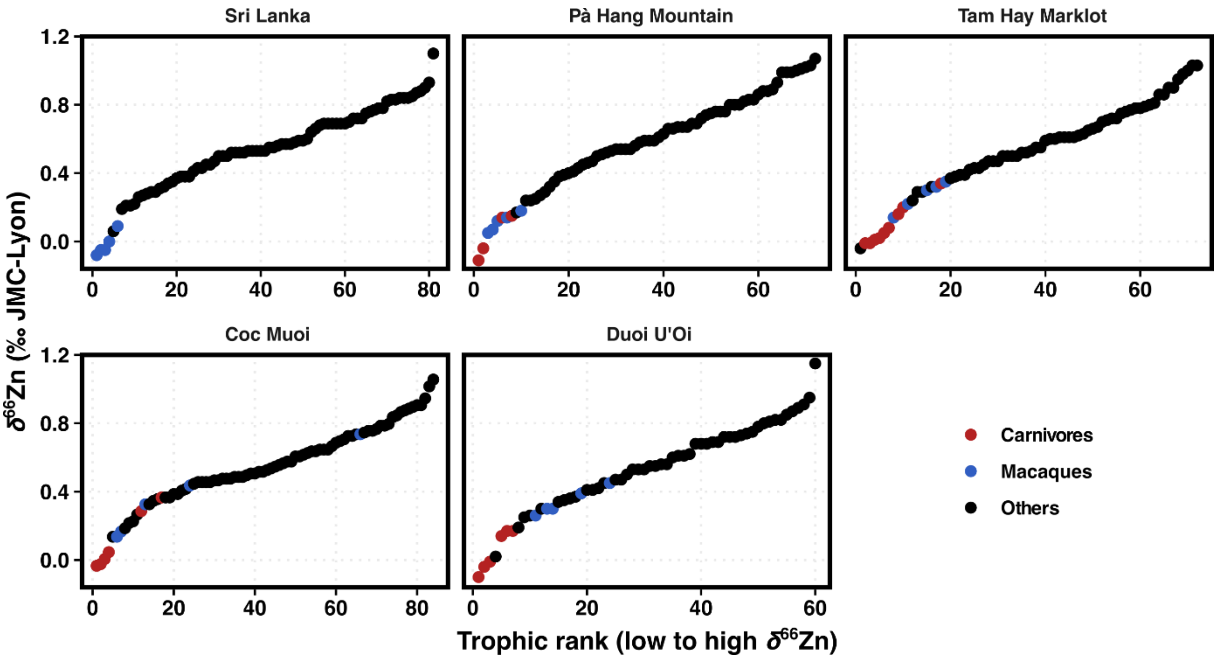

Supplementary Figure 8 | Rank-ordered δ^{66}Zn values and food web structure across Southeast Asian fossil assemblages. Rank-order plots of δ^{66}Zn values for fossil taxa from five tropical Southeast Asian assemblages (Sri Lanka, n = 81; Pà Hang Mountain, n = 76; Tam Hay Marklot, n = 72; Coc Muoi, n = 84; and Duoi U'Oi, n = 60). Despite ecological and taxonomic variation, all sites exhibit a comparable internal isotopic range and structure. In most sites, the lowest δ^{66}Zn ranks are occupied by carnivores (red), reflecting their high trophic position. At Sri Lanka, however, where obligate carnivores are absent, macaques (blue) occupy the lowest δ^{66}Zn ranks instead. This substitution, coupled with the consistency in overall isotopic structure, supports the interpretation of Sri Lankan macaques as occupying a trophic role analogous to that of carnivores in other assemblages.

Though limited behavioural studies exist for fossil macaques in Sri Lanka [1,52,53], numerous modern studies across Southeast Asia confirm that extant macaque species routinely incorporate animal matter in their diets, including insects, amphibians, bird eggs, and small mammals. This dietary flexibility is especially pronounced in tropical forest environments, where fallback foods often include invertebrate protein. The isotope data are thus ecologically plausible and consistent with macaques functioning as facultative omnivores or opportunistic carnivores, especially in depauperate or isolated ecosystems.

Taken together, the isotopic evidence from δ^{66}Zn values, Δ^{66}Zn spacing, bootstrapped confidence intervals, and rank-order distributions strongly support the interpretation that macaques in the Sri Lankan fossil foodweb occupied a higher trophic position than their herbivore and omnivore counterparts. Their δ^{66}Zn values are best understood as being indicative of a diet enriched in animal matter, consistent with behaviours observed in extant macaque populations [54,55] and analogous in isotopic signature to known carnivores in other assemblages [9–11], while representing a flexible and opportunistic feeding niche that contributed to the ecological resilience of the taxon in tropical environments.

Interpreting trophic position using δ^{66}Zn also requires consideration not only of the isotopic behaviour of Zn during trophic transfers, but also of differences in Zn concentration and bioavailability among

food resources. These factors influence the isotopic composition of assimilated Zn and can shift δ^{66}Zn values independently of large changes in overall dietary proportions. Notably, unlike δ^{15}N where a trophic-level signal can emerge with animal-protein contributions as low as ~20% [56–58], δ^{66}Zn has been shown to respond in a near-linear fashion to the relative proportions of plant versus animal material in the diet. Mass-balance experiments [19] and empirical datasets from archaeological ecosystems [11] consistently demonstrate this pattern. However, animal-derived foods typically contain substantially higher Zn concentrations than plant resources [33–35] and promote more efficient Zn absorption during digestion [28,29]. Because δ^{66}Zn values in consumers reflect the isotopic composition of *assimilated* rather than simply *ingested* Zn, even moderate incorporation of such high-Zn, highly bioavailable foods can exert a disproportionate influence on the resulting enamel δ^{66}Zn values. This effect is particularly relevant when interpreting "carnivore-like" δ^{66}Zn values in taxa that are not obligate carnivores. A consumer may obtain a relatively small proportion of its caloric intake from animal resources while still extracting a large proportion of its Zn from those same foods. As a result, δ^{66}Zn values could shift toward the carnivore range even in the absence of a mostly carnivorous diet.

In the absence of obligate carnivores in the Sri Lankan assemblage, the δ^{66}Zn values of macaques can serve as a reliable proxy for the higher end of the trophic spectrum. Isotopically, they exhibit all the hallmarks of a high trophic position: their δ^{66}Zn values are consistently lower than those of herbivores and omnivores at the same site, align closely with those of known carnivores from comparable tropical contexts, and occupy the lowest isotopic ranks within the foodweb. As such, macaques in Sri Lanka effectively anchors the upper trophic range of the assemblage, not due to ecological convention, but through the internal logic of the δ^{66}Zn isotopic structure itself.

Assigning dietary categories to the Sri Lankan assemblage

To evaluate the dietary position of the Sri Lankan fauna, we applied a univariate probabilistic classification framework based on zinc isotope values (δ^{66}Zn). While additional isotopic systems (δ^{13}C, δ^{18}O) provide valuable ecological context, they are influenced by a range of confounding factors in tropical rainforest environments, including canopy effects, C_3/C_4 mosaic vegetation, and heterogeneous feeding strategies (e.g., grazing, browsing, frugivory, varying degrees of omnivory). Because of these complexities, we restricted our classification exercise to δ^{66}Zn, which directly reflects trophic position and is less sensitive to local habitat structure.

This classification approach is enabled by the broadly consistent structure of the foodweb across sites when examined solely through δ^{66}Zn values. As outlined above, the comparative faunas exhibit highly similar internal ranges and distributions (**Supplementary Supplementary Figure *4***), likely shaped by tropical rainforest environments with broadly overlapping ecological parameters. In particular, the

isotopic spacing between taxa with the lowest and highest δ^{66}Zn values (**Supplementary Supplementary Figure *5***), as well as the characteristic shape of rank-order plots (**Supplementary Supplementary Figure *8***), demonstrates a reproducible trophic pattern across regions. This structural similarity justifies the transfer of dietary reference distributions from the mainland sites to the Sri Lankan assemblage.

As reference distributions, we used specimens from four mainland Southeast Asian localities (Pà Hang Mountain, Tam Hay Marklot, Coc Muoi, and Duoi U'Oi; [9–11]), where dietary assignments are well constrained by ecological and comparative data. For each major dietary class (Carnivore, Omnivore, Herbivore), we estimated kernel density functions of δ^{66}Zn values. Class priors were set to equal probability to avoid inflating the influence of groups with greater sample sizes. Bandwidths were selected via leave-one-site-out cross-validation, such that each site in turn was excluded from model fitting and then used for validation. This procedure optimized predictive performance across localities and limited overfitting to any single site. For each Sri Lankan specimen, we computed the posterior probability of membership in each dietary class by combining class-conditional kernel densities with these priors. Individuals were assigned to the dietary category with the highest posterior probability, and we also retained the maximum posterior as a measure of classification confidence.

For the training dataset, we conservatively categorized *Sus* sp. and *Pongo* sp. as herbivores rather than omnivores. Although both taxa can exhibit omnivorous feeding, published literature emphasizes that their predominant ecological role is herbivorous [59–64]. Aligning their classification with this behavioural tendency reduces ambiguity in the training set and results in more conservative dietary reference bands for subsequent prediction.

This approach yielded classifications consistent with expectations for several taxa. For instance, *Macaca sinica* from Sri Lanka was assigned to the carnivore category, paralleling our observation that its δ^{66}Zn values occupy the trophic range otherwise filled by carnivores at other sites. This result reinforces the interpretation that *M. sinica* maintained a higher trophic position, likely reflecting dietary reliance on high-trophic-level items such as invertebrates and small vertebrates. The dietary assignments derived from this procedure also reveal a nuanced structure among the Sri Lankan primates and humans. *Semnopithecus priam* and *Trachypithecus vetulus* are primarily classified as omnivores, although several individuals are assigned to the herbivore category, consistent with their mixed folivorous–frugivorous feeding ecology [65,66]. Humans are predominantly classified as omnivores (n = 14), with two individuals assigned to the carnivore category, and eight classified as herbivores. Notably, most of the herbivore-classified humans derive from younger periods, suggesting a temporal shift toward greater plant consumption. More broadly, the kernel-based predictions provide a

transparent, distribution-driven framework for situating the Sri Lankan fauna within dietary categories established at mainland sites, while quantifying the uncertainty inherent in such assignments.

Supplementary Material – Text 3: Relationship between δ^{66}Zn and δ^{13}C/δ^{18}O in Sri Lankan hunter-gatherers

In order to explore complementary aspects of Sri Lankan hunter-gatherers' ecology, we evaluate whether temporal variation in δ^{66}Zn covaries with other isotope data available for the same specimens. Notably, δ^{13}C reflects the isotopic composition of dietary carbon and, in tropical settings such as Sri Lanka, is sensitive to the structure of plant community [67], and δ^{18}O is influenced by ingested water and local hydroclimate, and can therefore track broader shifts in water sources and climatic conditions [68]. In principle, these proxies could be thus used to assess whether the gradual increase over time in δ^{66}Zn values observed in human specimens, consistent with greater reliance on plant resources, coincide with a shift in δ^{13}C toward specific plant communities (e.g., greater use of canopy-derived resources or forest edge/mosaics), or with δ^{18}O changes consistent with different water-use behaviours or hydroclimatic regimes. Changes in δ^{18}O can also vary among plant organs [68] and across canopy heights [69] and could thus reflect shifts in the types of plant tissues consumed [70], although disentangling dietary effects from hydroclimate-driven shifts could be challenging.

Such covariation with δ^{13}C and δ^{18}O values could help assess if trophic change occurred alongside broader changes in habitat use and resource procurement, potentially reflecting shifts in foraging territories, targeting of specific plant communities, or changing strategies of landscape use within rainforest environments (without necessarily implying land-cover change). Conversely, weak or absent covariation would support the interpretation that δ^{66}Zn captures trophic reorganization that is largely independent of the habitat and hydroclimatic information recorded by δ^{13}C and δ^{18}O. However, given uneven sample sizes among age groups and partial site–age confounding, these analyses are treated as exploratory and are not used as the primary basis for inference.

To explore whether variation in human enamel δ^{66}Zn covaries with enamel δ^{13}C and δ^{18}O, we fitted individual-level linear models of the form $\delta^{66}\text{Zn} \sim \delta^{13}\text{C} + \delta^{18}\text{O}$ (**Supplementary Table 3**). Human specimens (n = 24) are unevenly distributed across five periods (20–17 ka: n = 1; 17–16 ka: n = 7; 10 ka: n = 3; 7 ka: n = 9; 3 ka: n = 4), and one (17–16 ka) derives exclusively from a different site (Fa-Hien Lena) than the remaining groups (Balangoda Kuragala). Because such imbalance can cause regression estimates to be dominated by the largest group(s), we present both ordinary least squares (OLS) fits and weighted least squares (WLS) fits in which each period contributes equally (weights = 1/n within period).

Model ID	Model formula	Fit type	*n*	Notes	β(δ^{13}C)	*p*(δ^{13}C)	β(δ^{18}O)	*p*(δ^{18}O)	Adj. R^2	Model *p*
M1	Zn ~ C + O	OLS	24	Individual-level	-0.025	0.15	+0.058	0.06	0.104	0.12
M2	Zn ~ C + O	WLS	24	Age-balanced	-0.031	0.02	+0.064	0.001	0.351	0.004
M3	Zn ~ C + O + Site	OLS	24	Site partially confounded	-0.028	0.11	+0.054	0.08	0.147	0.11
M4	Zn ~ C + O + Site	WLS	24	Age-balanced + Site	-0.035	0.012	+0.069	0.0007	0.398	0.004
M5	Zn ~ C + O	OLS	23	Excluding 20–17 ka	-0.025	0.17	+0.054	0.21	0.039	0.26
M6	Zn ~ C + O	WLS	23	Excluding 20–17 ka	-0.030	0.04	+0.046	0.24	0.127	0.10
M7	Zn ~ C + O	OLS	17	Excluding 17–16 ka	-0.033	0.06	+0.055	0.08	0.200	0.08
M8	Zn ~ C + O	WLS	17	Excluding 17–16 ka	-0.037	0.01	+0.070	0.001	0.510	0.003

Supplementary Table 3 | Summary of regression models relating δ^{66}Zn to δ^{13}C and δ^{18}O. Ordinary least squares (OLS) and age-balanced weighted least squares (WLS; 1/*n*) models testing associations between enamel δ^{66}Zn (Zn) and δ^{13}C/δ^{18}O (C and O).

In the unweighted OLS model, δ^{66}Zn shows only weak evidence of association with δ^{13}C and δ^{18}O (R^2 = 0.18; overall p = 0.12), with a positive almost-significant δ^{18}O slope (p = 0.06) and a negative but non-significant δ^{13}C slope (p = 0.15). In contrast, the age-balanced WLS model indicates a negative association between δ^{66}Zn and δ^{13}C (estimate = −0.031; p = 0.02) and a positive association between δ^{66}Zn and δ^{18}O (estimate = +0.065; p = 0.001), with improved fit (Adj. R^2 = 0.35; overall p = 0.004). Including site as an additional covariate produced similar coefficients, although site effects are difficult to interpret because site is partially confounded with age group (17–16 ka).

We performed sensitivity checks to assess robustness. Excluding the single observation in the 20–17 ka group (n = 1) reduced support for the δ^{18}O effect in the WLS model (p = 0.24), while the negative δ^{13}C association remained (p = 0.04). Conversely, excluding the site-confounded 17–16 ka group strengthened the WLS associations for both δ^{13}C and δ^{18}O (p = 0.0095 and p = 0.001, respectively). Leave-one-out refits further indicate that coefficient estimates can shift with the removal of individual observations, consistent with the small sample size in some age groups (notably 10 ka, n = 3) and the presence of influential points.

Overall, these analyses suggest potential covariation between δ^{66}Zn and δ^{13}C/δ^{18}O, particularly when age groups are weighted equally to mitigate uneven sample sizes, which could indicate that trophic shifts inferred from δ^{66}Zn occurred alongside broader changes in habitat use and/or water and plant-resource procurement strategies. However, inference is limited by 1) strong imbalance in group sizes, including one group represented by a single specimen, 2) partial confounding between age group and site, and 3) sensitivity to influential observations in small groups. We therefore treat these relationships as exploratory and do not rely on them for primary interpretation.

Reference

1. Roberts, P. *et al.* Fruits of the forest: Human stable isotope ecology and rainforest adaptations in Late Pleistocene and Holocene (~36 to 3 ka) Sri Lanka. *J. Hum. Evol.* **106**, 102–118 (2017).
2. Hillson, S. *Dental Anthropology*. (Cambridge University Press, Cambridge, 1996).
3. AlQahtani, S. J., Hector, M. P. & Liversidge, H. M. Brief communication: The London atlas of human tooth development and eruption. *Am. J. Phys. Anthropol.* **142**, 481–490 (2010).
4. Roberts, P. *et al.* Direct evidence for human reliance on rainforest resources in late Pleistocene Sri Lanka. *Science* **347**, 1246–1249 (2015).
5. Amano, N. *et al.* Of forests and grasslands: human, primate, and ungulate palaeoecology in Late Pleistocene-Holocene Sri Lanka. *Front. Earth Sci.* **11**, (2023).
6. Jaouen, K., Pons, M.-L. & Balter, V. Iron, copper and zinc isotopic fractionation up mammal trophic chains. *Earth Planet. Sci. Lett.* **374**, 164–172 (2013).
7. Jaouen, K., Beasley, M., Schoeninger, M., Hublin, J.-J. & Richards, M. P. Zinc isotope ratios of bones and teeth as new dietary indicators: Results from a modern food web (Koobi Fora, Kenya). *Sci. Rep.* **6**, srep26281 (2016).
8. Jaouen, K., Szpak, P. & Richards, M. P. Zinc isotope ratios as indicators of diet and trophic level in arctic marine mammals. *PLoS ONE* **11**, e0152299 (2016).
9. Bourgon, N. *et al.* Zinc isotopes in Late Pleistocene fossil teeth from a Southeast Asian cave setting preserve paleodietary information. *Proc. Natl. Acad. Sci. U.S.A.* **117**, 4675–4681 (2020).
10. Bourgon, N. *et al.* Trophic ecology of a Late Pleistocene early modern human from tropical Southeast Asia inferred from zinc isotopes. *J. Hum. Evol.* **161**, 103075 (2021).
11. Bourgon, N. *et al.* Faunal persistence and ecological flexibility in Pleistocene Southeast Asia revealed through multi-isotope analysis. *Sci. Adv.* **11**, eadu3642 (2025).
12. McCormack, J. *et al.* Trophic position of *Otodus megalodon* and great white sharks through time revealed by zinc isotopes. *Nat. Commun.* **13**, 2980 (2022).
13. McCormack, J. *et al.* Zinc isotopes from archaeological bones provide reliable trophic level information for marine mammals. *Commun. Biol.* **4**, 1–11 (2021).
14. McCormack, J., Karnes, M., Haulsee, D., Fox, D. & Kim, S. L. Shark teeth zinc isotope values document intrapopulation foraging differences related to ontogeny and sex. *Commun. Biol.* **6**, 1–10 (2023).
15. Guiserix, D. *et al.* Stable isotope composition and concentration systematics of Ca and trace elements (Zn, Sr) in single aliquots of fossil bone and enamel. *Geochim. Cosmochim. Acta* **367**, 123–132 (2024).
16. Pederzani, S. *et al.* Stable isotopes show *Homo sapiens* dispersed into cold steppes ~45,000 years ago at Ilsenhöhle in Ranis, Germany. *Nat. Ecol. Evol.* **8**, 578–588 (2024).
17. Dean, C., Le Cabec, A., Spiers, K., Zhang, Y. & Garrevoet, J. Incremental distribution of strontium and zinc in great ape and fossil hominin cementum using synchrotron X-ray fluorescence mapping. *J. R. Soc. Interface* **15**, 20170626 (2018).
18. McCormack, J. *et al.* Zinc isotope composition of enameloid, bone and muscle of gilt-head seabreams (*Sparus aurata*) raised in pisciculture and their relation to diet. *Mar. Biol.* **171**, 65 (2024).

19. Bourgon, N. *et al.* Dietary and homeostatic controls of Zn isotopes in rats: A controlled feeding experiment and modeling approach. *Metallomics* **16**, mfae026 (2024).

20. Balter, V. *et al.* Contrasting Cu, Fe, and Zn isotopic patterns in organs and body fluids of mice and sheep, with emphasis on cellular fractionation. *Metallomics* **5**, 1470–1482 (2013).

21. Moynier, F., Fujii, T., S. Shaw, A. & Borgne, M. L. Heterogeneous distribution of natural zinc isotopes in mice. *Metallomics* **5**, 693–699 (2013).

22. Mahan, B., Moynier, F., Jørgensen, A. L., Habekost, M. & Siebert, J. Examining the homeostatic distribution of metals and Zn isotopes in Göttingen minipigs. *Metallomics* **10**, 1264–1281 (2018).

23. Brown, K. H. *et al.* International Zinc Nutrition Consultative Group (IZiNCG) technical document #1: Assessment of the risk of zinc deficiency in populations and options for its control. *Food Nutr. Bull.* **25**, S99-203 (2004).

24. Turnlund, J. R., King, J. C., Keyes, W. R., Gong, B. & Michel, M. C. A stable isotope study of zinc absorption in young men: Effects of phytate and a-cellulose. *Am. J. Clin. Nutr.* **40**, 1071–1077 (1984).

25. Ferguson, E. L., Gibson, R. S., Thompson, L. U., Ounpuu, S. & Berry, M. Phytate, zinc, and calcium contents of 30 East African foods and their calculated phytate:Zn, Ca:phytate, and [Ca][phytate]/[Zn] molar ratios. *J. Food Compost. Anal.* **1**, 316–325 (1988).

26. Kristensen, M. B. *et al.* Total zinc absorption in young women, but not fractional zinc absorption, differs between vegetarian and meat-based diets with equal phytic acid content. *Br. J. Nutr.* **95**, 963–967 (2006).

27. Foster, M. & Samman, S. Vegetarian diets across the lifecycle: Impact on zinc intake and status. *Adv. Food Nutr. Res.* **74**, 93–131 (2015).

28. Sandström, B., Arvidsson, B., Cederblad, A. & Björn-Rasmussen, E. Zinc absorption from composite meals: The significance of wheat extraction rate, zinc, calcium, and protein content in meals based on bread. *Am. J. Clin. Nutr.* **33**, 739–745 (1980).

29. Wapnir, R. A. Zinc deficiency, malnutrition and the gastrointestinal tract. *J. Nutr.* **130**, 1388S-1392S (2000).

30. Sandström, B., Cederblad, Å. & Lönnerdal, B. Zinc absorption from human milk, cow's milk, and infant formulas. *Am. J. Dis. Child.* **137**, 726–729 (1983).

31. Sandström, B., Almgren, A., Kivistö, B. & Cederblad, Å. Effect of protein level and protein source on zinc absorption in humans. *J. Nutr.* **119**, 48–53 (1989).

32. Davidsson, L., Almgren, A., Sandström, B., Juillerat, M. E.-A. & Hurrell, R. F. Zinc absorption in adult humans: The effect of protein sources added to liquid test meals. *Br. J. Nutr.* **75**, 607–613 (1996).

33. Onianwa, P. C., Adeyemo, A. O., Idowu, O. E. & Ogabiela, E. E. Copper and zinc contents of Nigerian foods and estimates of the adult dietary intakes. *Food Chem.* **72**, 89–95 (2001).

34. Scherz, H. & Kirchhoff, E. Trace elements in foods: Zinc contents of raw foods—A comparison of data originating from different geographical regions of the world. *J. Food Compost. Anal.* **19**, 420–433 (2006).

35. Reddy, N. R. Occurrence, distribution, content, and dietary intake of phytate. in *Food phytates* (eds Reddy, N. R. & Sathe, S. K.) 37–63 (CRC Press, Boca Raton, 2002).

36. Opfergelt, S. *et al.* The influence of weathering and soil organic matter on Zn isotopes in soils. *Chem. Geol.* **466**, 140–148 (2017).

37. Liang, B., Han, G. & Zhao, Y. Zinc isotopic signature in tropical soils: A review. *Sci. Total Environ.* **820**, 153303 (2022).

38. Junqueira, T. P. *et al.* Applications of zinc stable isotope analysis in environmental and biological systems: a review. *Geochem.: Explor., Environ., Anal.* **24**, geochem2024-003 (2024).

39. Viers, J. *et al.* Evidence of Zn isotopic fractionation in a soil–plant system of a pristine tropical watershed (Nsimi, Cameroon). *Chem. Geol.* **239**, 124–137 (2007).

40. Little, S. H. *et al.* Cu and Zn isotope fractionation during extreme chemical weathering. *Geochim. Cosmochim. Acta.* **263**, 85–107 (2019).

41. Aucour, A.-M. *et al.* Dynamics of Zn in an urban wetland soil–plant system: Coupling isotopic and EXAFS approaches. *Geochim. Cosmochim. Acta* **160**, 55–69 (2015).

42. Jouvin, D. *et al.* Stable isotopes of Cu and Zn in higher plants: Evidence for Cu reduction at the root surface and two conceptual models for isotopic fractionation processes. *Environ. Sci. Technol.* **46**, 2652–2660 (2012).

43. Wiggenhauser, M. *et al.* Zinc isotope fractionation during grain filling of wheat and a comparison of zinc and cadmium isotope ratios in identical soil–plant systems. *New Phytol.* **219**, 195–205 (2018).

44. Weiss, D. J. *et al.* Isotopic discrimination of zinc in higher plants. *New Phytol.* **165**, 703–710 (2005).

45. Moynier, F. *et al.* Isotopic fractionation and transport mechanisms of Zn in plants. *Chem. Geol.* **267**, 125–130 (2009).

46. Aucour, A. M., Pichat, S., Macnair, M. R. & Oger, P. Fractionation of stable zinc isotopes in the zinc hyperaccumulator *Arabidopsis halleri* and nonaccumulator *Arabidopsis petraea*. *Environ. Sci. Technol.* **45**, 9212–9217 (2011).

47. Tang, Y.-T. *et al.* Zinc isotope fractionation in the hyperaccumulator *Noccaea caerulescens* and the nonaccumulating plant *Thlaspi arvense* at low and high Zn supply. *Environ. Sci. Technol.* **50**, 8020–8027 (2016).

48. van der Ent, A. *et al.* Isotopic signatures reveal zinc cycling in the natural habitat of hyperaccumulator *Dichapetalum gelonioides* subspecies from Malaysian Borneo. *BMC Plant Biol.* **21**, 437 (2021).

49. Jaouen, K. *et al.* A Neandertal dietary conundrum: Insights provided by tooth enamel Zn isotopes from Gabasa, Spain. *Proc. Natl. Acad. Sci. U.S.A.* **119**, e2109315119 (2022).

50. Bacon, A.-M. *et al.* Late Pleistocene mammalian assemblages of Southeast Asia: New dating, mortality profiles and evolution of the predator–prey relationships in an environmental context. *Palaeogeogr. Palaeoclimatol. Palaeoecol.* **422**, 101–127 (2015).

51. Bacon, A.-M. *et al.* A multi-proxy approach to exploring *Homo sapiens*' arrival, environments and adaptations in Southeast Asia. *Sci. Rep.* **11**, 21080 (2021).

52. Dittus, W. P. J. Toque macaque food calls: Semantic communication concerning food distribution in the environment. *Anim. Behav.* **32**, 470–477 (1984).

53. Nekaris, K. A. I. & Silva Wijeyeratne, G. de. *The Primates of Sri Lanka*. (Sri Lanka Tourism Promotion Bureau, Colombo [Sri Lanka], 2009).

54. Fooden, J. Taxonomy and evolution of the *sinica* group of macaques: I. Species and subspecies accounts of *Macaca sinica*. *Primates* **20**, 109–140 (1979).

55. Wijesooriya, K., Weerasekara, L. & Ranawana, K. Agamid lizard predation by *Macaca sinica* (toque macaque) in Peradeniya, Sri Lanka. *Mammalia* **86**, 463–467 (2022).

56. Ambrose, S. H. Controlled diet and climate experiments on nitrogen isotope ratios of rats. in *Biogeochemical approaches to paleodietary analysis* (eds Ambrose, S. H. & Katzenberg, M. A.) vol. 5 243–259 (Springer, Boston, 2002).

57. Sponheimer, M. *et al.* Nitrogen isotopes in mammalian herbivores: Hair δ^{15}N values from a controlled feeding study. *Int. J. Osteoarchaeol.* **13**, 80–87 (2003).

58. Leichliter, J. N. *et al.* Nitrogen isotopes in tooth enamel record diet and trophic level enrichment: Results from a controlled feeding experiment. *Chem. Geol.* **563**, 120047 (2021).

59. Barrios-Garcia, M. N. & Ballari, S. A. Impact of wild boar (*Sus scrofa*) in its introduced and native range: A review. *Biol. Invasions* **14**, 2283–2300 (2012).

60. Ballari, S. A. & Barrios-García, M. N. A review of wild boar *Sus scrofa* diet and factors affecting food selection in native and introduced ranges. *Mamm. Rev.* **44**, 124–134 (2014).

61. Fox, E. A., Schaik, C. P. van, Sitompul, A. & Wright, D. N. Intra-and interpopulational differences in orangutan (*Pongo pygmaeus*) activity and diet: Implications for the invention of tool use. *Am. J. Phys. Anthropol.* **125**, 162–174 (2004).

62. Wich, S. A., Utami-Atmoko, S. S., Mitra Setia, T., Djoyosudharmo, S. & Geurts, M. L. Dietary and energetic responses of *Pongo abelii* to fruit availability fluctuations. *Int. J. Primatol.* **27**, 1535–1550 (2006).

63. Kanamori, T., Kuze, N., Bernard, H., Malim, T. P. & Kohshima, S. Feeding ecology of Bornean orangutans (*Pongo pygmaeus morio*) in Danum Valley, Sabah, Malaysia: A 3-year record including two mast fruitings. *Am. J. Primatol.* **72**, 820–840 (2010).

64. Hardus, M. E. *et al.* Behavioral, ecological, and evolutionary aspects of meat-eating by Sumatran orangutans (*Pongo abelii*). *Int. J. Primatol.* **33**, 287–304 (2012).

65. Vandercone, R. P., Dinadh, C., Wijethunga, G., Ranawana, K. & Rasmussen, D. T. Dietary diversity and food selection in Hanuman langurs (*Semnopithecus entellus*) and purple-faced langurs (*Trachypithecus vetulus*) in the Kaludiyapokuna Forest Reserve in the dry zone of Sri Lanka. *Int. J. Primatol.* **33**, 1382–1405 (2012).

66. Vanaraj, G. & Pragasan, L. A. Activity and dietary budgets of Tufted grey langurs (*Semnopithecus priam priam*). *Ethol. Ecol. Evol.* **33**, 477–495 (2021).

67. Farquhar, G. D., Ehleringer, J. R. & Hubick, K. T. Carbon isotope discrimination and photosynthesis. *Annu. Rev. Plant Physiol. Plant Mol. Biol.* **40**, 503–537 (1989).

68. Pederzani, S. & Britton, K. Oxygen isotopes in bioarchaeology: Principles and applications, challenges and opportunities. *Earth Sci. Rev.* **188**, 77–107 (2019).

69. van der Merwe, N. J. & Medina, E. The canopy effect, carbon isotope ratios and foodwebs in Amazonia. *J. Archaeol. Sci.* **18**, 249–259 (1991).

70. Roberts, P., Blumenthal, S. A., Dittus, W., Wedage, O. & Lee-Thorp, J. A. Stable carbon, oxygen, and nitrogen, isotope analysis of plants from a South Asian tropical forest: Implications for primatology. *Am. J. Primatol.* **79**, e22656 (2017).
