# Supplementary material for: Pre-agricultural intensification of plant use in Pleistocene Sri Lankan rainforests
Source: Nat Ecol Evol. 2026 May 20;10(7):1245–53. doi: 10.1038/s41559-026-03082-6 (PMC13345896; doi:10.1038/s41559-026-03082-6)
Supplement: Supplementary file 2 — Reporting summary [file 41559_2026_3082_MOESM2_ESM.pdf]

nature portfolio

Corresponding author(s): Nicolas Bourgon

Last updated by author(s): Mar 13, 2026

Reporting Summary

Nature Portfolio wishes to improve the reproducibility of the work that we publish. This form provides structure for consistency and transparency in reporting. For further information on Nature Portfolio policies, see our Editorial Policies and the Editorial Policy Checklist.

Statistics

For all statistical analyses, confirm that the following items are present in the figure legend, table legend, main text, or Methods section.

n/a	Confirmed	
☐	☒	The exact sample size (*n*) for each experimental group/condition, given as a discrete number and unit of measurement
☐	☒	A statement on whether measurements were taken from distinct samples or whether the same sample was measured repeatedly
☐	☒	The statistical test(s) used AND whether they are one- or two-sided<br>*Only common tests should be described solely by name; describe more complex techniques in the Methods section.*
☐	☒	A description of all covariates tested
☐	☒	A description of any assumptions or corrections, such as tests of normality and adjustment for multiple comparisons
☐	☒	A full description of the statistical parameters including central tendency (e.g. means) or other basic estimates (e.g. regression coefficient) AND variation (e.g. standard deviation) or associated estimates of uncertainty (e.g. confidence intervals)
☐	☒	For null hypothesis testing, the test statistic (e.g. *F*, *t*, *r*) with confidence intervals, effect sizes, degrees of freedom and *P* value noted<br>*Give P values as exact values whenever suitable.*
☒	☐	For Bayesian analysis, information on the choice of priors and Markov chain Monte Carlo settings
☒	☐	For hierarchical and complex designs, identification of the appropriate level for tests and full reporting of outcomes
☒	☐	Estimates of effect sizes (e.g. Cohen's *d*, Pearson's *r*), indicating how they were calculated

Our web collection on statistics for biologists contains articles on many of the points above.

Software and code

Policy information about availability of computer code

Data collection	n/a
Data analysis	Open-source program R software (R version 4.5.1) and R packages ggplot2, tidyverse, cowplot, and rstatix.

For manuscripts utilizing custom algorithms or software that are central to the research but not yet described in published literature, software must be made available to editors and reviewers. We strongly encourage code deposition in a community repository (e.g. GitHub). See the Nature Portfolio guidelines for submitting code & software for further information.

Data

Policy information about availability of data

All manuscripts must include a data availability statement. This statement should provide the following information, where applicable:

- Accession codes, unique identifiers, or web links for publicly available datasets
- A description of any restrictions on data availability
- For clinical datasets or third party data, please ensure that the statement adheres to our policy

All new δ^{66}Zn data produced for this study are included in the Supplementary Information. Previously published comparative datasets from Pà Hang Mountain, Tam Hay Marklot, Coc Muoi, and Duoi U'Oi can be accessed through the original publications cited in the manuscript.

Research involving human participants, their data, or biological material

Policy information about studies with human participants or human data. See also policy information about sex, gender (identity/presentation), and sexual orientation and race, ethnicity and racism.

Reporting on sex and gender	n/a
Reporting on race, ethnicity, or other socially relevant groupings	n/a
Population characteristics	n/a
Recruitment	n/a
Ethics oversight	n/a

Note that full information on the approval of the study protocol must also be provided in the manuscript.

Field-specific reporting

Please select the one below that is the best fit for your research. If you are not sure, read the appropriate sections before making your selection.

☐ Life sciences ☐ Behavioural & social sciences ☒ Ecological, evolutionary & environmental sciences

For a reference copy of the document with all sections, see nature.com/documents/nr-reporting-summary-flat.pdf

Ecological, evolutionary & environmental sciences study design

All studies must disclose on these points even when the disclosure is negative.

Study description	This study investigates long-term dietary change among Late Pleistocene–Holocene humans in Sri Lanka using zinc isotope analysis of tooth enamel.
Research sample	The dataset includes enamel samples from 24 human individuals and 57 faunal specimens recovered from three Sri Lankan cave sites (Balangoda Kuragala, Batadomba-lena, and Fa-Hien Lena).
Sampling strategy	No new sampling was conducted; all analyses used residual enamel powders from previous studies (Roberts et al., 2015, 2017; Amano et al., 2023). Specimens were selected based on sufficient remaining material and, for the faunal dataset, the availability (where possible) of at least five individuals per taxon.
Data collection	Zinc isotope measurements were generated using established multi-collector ICP-MS protocols by Nicolas Bourgon and Marcus Oelze
Timing and spatial scale	The samples span ~20–3 ka and derive from sites located within Sri Lanka's wet-zone rainforest, representing a single regional environmental context.
Data exclusions	No data exclusion
Reproducibility	Analytical reproducibility was ensured through repeated measurements of reference materials. Full reproducibility metrics are provided in the Supplementary Information.
Randomization	n/a
Blinding	n/a

Did the study involve field work? ☐ Yes ☒ No

Reporting for specific materials, systems and methods

We require information from authors about some types of materials, experimental systems and methods used in many studies. Here, indicate whether each material, system or method listed is relevant to your study. If you are not sure if a list item applies to your research, read the appropriate section before selecting a response.

Materials & experimental systems

n/a	Involved in the study	
☒	☐	Antibodies
☒	☐	Eukaryotic cell lines
☐	☒	Palaeontology and archaeology
☒	☐	Animals and other organisms
☒	☐	Clinical data
☒	☐	Dual use research of concern
☒	☐	Plants

Methods

n/a	Involved in the study	
☒	☐	ChIP-seq
☒	☐	Flow cytometry
☒	☐	MRI-based neuroimaging

Palaeontology and Archaeology

Specimen provenance: All specimens originate from collections held by the Department of Archaeology, Government of Sri Lanka. The original specimens from which enamel powders were derived are stored at the Department of Archaeology, Government of Sri Lanka. No new sampling was conducted for this study, and no additional permits were required.

Specimen deposition: No new sampling was conducted. All analyses were performed on residual enamel powders from previous studies (Roberts et al., 2015, 2017; Amano et al., 2023)

Dating methods: n/a

☐ Tick this box to confirm that the raw and calibrated dates are available in the paper or in Supplementary Information.

Ethics oversight: Ethical approval and guidelines were not applicable to this study, as it relied exclusively on previously collected fossil specimens. No work involving living animals or human participants was conducted.

Note that full information on the approval of the study protocol must also be provided in the manuscript.

Plants

Seed stocks: n/a

Novel plant genotypes: n/a

Authentication: n/a
